# Perioperative Low-Dose Ketamine for Postoperative Pain Management in Spine Surgery: A Systematic Review and Meta-Analysis of Randomized Controlled Trials

**DOI:** 10.1155/2022/1507097

**Published:** 2022-03-31

**Authors:** Lijin Zhou, Honghao Yang, Yong Hai, Yunzhong Cheng

**Affiliations:** Department of Orthopedic Surgery, Beijing Chao-Yang Hospital, Beijing, China

## Abstract

**Objective:**

Although low-dose ketamine has been shown to be generally beneficial in terms of pain control in a variety of major surgery, there is no consensus regarding the effectiveness of supplemental ketamine analgesic use exclusively in spine surgery. The objective of this systematic review and meta-analysis of randomized controlled trials (RCTs) was to assess the efficacy and safety of perioperative low-dose ketamine for pain management and analgesic consumption in patients undergoing spine surgery.

**Methods:**

A comprehensive literature search was performed for relevant studies using PubMed, EMBASE, Web of Science, and Cochrane Library. Patients who received perioperative low-dose ketamine were compared to the control group in terms of postoperative pain intensity, opioid consumption, and adverse events. Patients were further categorized by ages and administration times for subgroup analysis.

**Results:**

A total of 30 RCTs comprising 1,865 patients undergoing elective spine surgery were included. Significantly lower pain intensity and less opioid consumption at 12 h, 24 h, and 48 h postoperatively and lower incidence of postoperative nausea and vomiting (PONV) were observed in the ketamine group (all *P* < 0.05). There was no significant difference of central nervous system (CNS) adverse events between groups. However, different efficacy of low-dose ketamine was detected when patients were categorized by ages and administration times.

**Conclusion:**

Perioperative low-dose ketamine demonstrated analgesic and morphine-sparing effect with no increased adverse events after spine surgery. However, this effect was not significant in pediatric patients. Only postoperative or intraoperative and postoperative administration could prolong the analgesic time up to 48 h postoperatively. Further studies should focus on the optimal protocol of ketamine administration and its effect on old age participants.

## 1. Introduction

The postoperative pain is excessively difficult to management for patients undergoing various orthopedic surgery, particularly in spine surgery [1]. Inadequate postoperative pain control after spine surgery could impact patient well-being and rehabilitation, which remains a major clinical challenge for both spine surgeons and anesthesiologists [2].

It has been reported that spinal surgical procedures, especially in spinal fusion, always necessitate substantial pain control in the perioperative period [3]. To achieve satisfactory pain management, opioids have been the mainstay of analgesia after various spine surgery [4]. However, opioid-related adverse effects, including nausea and vomiting, pruritus, hallucination, nightmare, cardiovascular events, and even respiratory depression, frequently occurred in patients. [5–7] Also, the development of opioid-induced hyperalgesia (OIH) and/or acute opioid tolerance could consequently increase the postoperative opioid consumption and prolonged opioid-dependence that contribute substantially to the current opioid epidemic [8, 9].

Multimodal analgesia, which could achieve better postoperative pain control and reduce the need of opioid with concomitant reduction of opioid-related side effects through additive or synergistic effects of various nonopioids, has been widely reported as the leading principle of pain management after spine surgery [10–12]. Thus, finding optimal components of multimodal analgesia is of paramount importance.

Ketamine, a nonselective antagonist of N-methyl-D-aspartate (NMDA) receptor, has been proposed as a component of multimodal analgesia for various surgical procedures, as it could inhabit the pathway of central sensitization and secondary postoperative hyperalgesia [13]. At subanesthetic doses, ketamine is effective as an adjuvant medication to standard regimen of opioids, demonstrating prominent analgesic effect and opioid-sparing effect, with no unwanted side effects of the drug [14].

Although low-dose ketamine has been shown to be generally beneficial in terms of pain control in a variety of major surgery, there is no consensus regarding the effectiveness of supplemental ketamine analgesic use exclusively in spine surgery. Also, it is unclear how the ages of patients and administration time affect the effectiveness of ketamine in pain mitigating. The objective of this systematic review and meta-analysis of randomized controlled trials (RCTs) was to assess the efficacy and safety of perioperative low-dose ketamine for pain management and analgesic consumption in patients undergoing spine surgery.

## 2. Materials and Methods

This study was designed according to the Preferred Reporting Items for Systematic Reviews and Meta-Analyses (PRISMA) guidelines and registered with PROSPERO (ID: CRD42021238943) [15, 16]. As the data involved in this study are anonymized and freely available in the public domain, in which informed consent has already been obtained at the time of original data collection, this study is exempt from ethical approval.

### 2.1. Search Strategy

The PubMed, EMBASE, Web of Science, and Cochrane Library database were searched using the following terms: (Ketamine) AND (((((((((microdiscectomy) OR (discectomy)) OR (spine)) OR (spinal)) OR (scoliosis)) OR (disc)) OR (disk)) OR (lumbar)) OR (thoracic)).

The literature search was last updated on August 01, 2021. Two reviewers (L.Z. and H.Y.) independently screened the titles and abstracts, and any arising differences were settled by a discussion with a third party (Y.C.).

### 2.2. Inclusion Criteria

#### 2.2.1. Participants

Participants undergoing elective spine surgery were included in this study. We categorized participants as pediatric (10 to 17 years of age) and adult (≥18 years of age).

#### 2.2.2. Interventions

Patients received low-dose ketamine as

1. An intravenous (IV) bolus dose of 0.1–0.5 mg/kg intraoperatively
2. A continuous intravenous infusion of 1.0–10.0 *μ*g kg^−1^min^−1^ intraoperatively and terminated in 48 h after surgery
3. A dose of 0.1–1.0 mg/ml in intravenous patient-controlled analgesia (IV-PCA) devices postoperatively
4. Combination of an IV bolus, an infusion, and IV-PCA devices

Ketamine in combination with a basic analgesic regimen was acceptable if such interventions were administered in a same way to both intervention and control groups.

#### 2.2.3. Control

Control individuals comprise those who were administered an IV bolus, a continuous intravenous infusion, or IV-PCA of placebo or basic analgesic alone.

#### 2.2.4. Outcome Measures

*(1) Primary Outcomes.*

1. Pain intensity at rest and during mobilization using the Numerical Rating Scale (NRS) or Visual Analogue Scale (VAS) at 6 h, 12 h, 24 h, and 48 h postoperatively
2. Cumulative consumption of opioids in milligrams of morphine equivalents in the first 12 h, 24 h, and 48 h postoperatively, administrated by IV-PCA devices or as rescue medication

*(2) Secondary Outcomes.*

1. Time to first request for analgesia (trigger of IV-PCA) after surgery
2. The incidence of postoperative central nervous system (CNS) adverse events and postoperative nausea and vomiting (PONV)

#### 2.2.5. Study Design

Only the studies that described a prospective RCT were included.

### 2.3. Exclusion Criteria

Studies that met any of the following exclusion criteria were excluded: (1) patients not undergoing general anesthesia; (2) postoperative pain intensity or consumption of opioids was not reported; (3) reviews, case reports, and animal research; (4) duplicated publications; or (5) articles not published in English.

### 2.4. Assessment of Study Quality

The study quality was assessed independently by two reviewers (L.Z. and H.Y.) according to the Cochrane Handbook version 5.2.0 [17]. The specific contents of the assessment included random sequence generation, allocation of concealment, blinding, incomplete outcome data, selective outcome reporting, and other bias.

The level of certainty for the results was evaluated using the guidelines of the Recommendations, Assessment, Development, and Evaluation (GRADE) system [18]. The five domains included risk of bias, inconsistency, indirectness, imprecision, and publication bias. The level of certainty was graded using GRADEpro GDT online tool [19].

### 2.5. Data Extraction

Data extraction was performed by two reviewers independently (H.Y. and J.L.). The following study characteristics were recorded: demographic information, general anesthetic, bolus dosage, infusion dosage, timing of ketamine administration, interventions of control group, and primary postoperative analgesic. Continuous outcomes included pain intensity, cumulative consumption of opioids, and the time from end of surgery to first request for analgesia or first trigger of IV-PCA. Dichotomous outcomes included the postoperative CNS adverse events and PONV. Outcomes reported by at least five studies would be analyzed. The graphed data were interpolated using the tool WebPlotDigitizer.

### 2.6. Data Normalization

Extracted data were normalized prior to analysis. Pain intensity was assessed using various pain scores (0 = no pain) by the included studies, including Visual Analogue Scale (VAS) of 0 to 10, a Numerical Rating Scale (NRS) of 0 to 10, or a Verbal Rating Scale (VRS) of 0 to 5. We multiplied each pain score by 10 or 25 to convert them to a VAS of 0 to 100 [20]. Opioid for postoperative analgesia was administered as morphine, fentanyl, oxycodone, or hydromorphone in the included studies. Therefore, we converted the postoperative opioid consumption to morphine equivalents using conversion equations, such as 100:1 for IV fentanyl:IV morphine, 2:3 for IV oxycodone:IV morphine, 1:5 for IV hydromorphone:IV morphine, and 1:2 for IV methadone:IV morphine [21, 22]. For studies that reported opioid consumption over select time periods (e.g., 0–24 h, 24–48 h), the mean of cumulative opioid consumption was calculated, and the standard deviations were estimated according to the Cochrane Handbook.

### 2.7. Data Synthesis and Statistical Analysis

For studies with multiple treatment arms, the arms that involved an intervention not defined by the inclusion criteria would be excluded, and the data in other arms would be combined to create a single pair-wise comparison as described by. All statistical analysis was performed using Stata 15.1. For continuous outcomes, the weighted mean difference (WMD) was utilized for estimating effect. The effect measure of dichotomous outcomes was displayed as a risk ratio (RR). Statistical heterogeneity among studies was evaluated using the I-square test and Cochran's Q test. If the *I*^2^ value was less than 50% and the *P* value was greater than 0.10, the fixed-effects model was performed; if the *I*^2^ value was greater than 50% or the *P* value was less than 0.10, the random-effects model would be used.

### 2.8. Subgroup Analysis

The included studies were categorized for subgroup analysis:

1. By ages: pediatric spine surgery vs. adult spine surgery
2. By administration times of ketamine: intraoperatively (intragroup) vs. postoperatively (postgroup) vs. intraoperatively and postoperatively (intragroup + postgroup)

We restricted the subgroup analysis to the primary outcome and adverse events. Each subgroup should include at least two studies. Subgroup analysis by administration times was only performed for studies about adult spine surgery. The results of subgroup analysis were available in Supplementary Materials.

### 2.9. Assessment of Publication Bias

Potential publication bias was assessed by the application of Egger's test at the *P* value less than 0.10 level of significance. If publication bias was indicated, we further evaluated the number of missing studies in this meta-analysis by the application of the trim and fill method and recalculated the pooled WMD or RR with the addition of those missing studies [23].

## 3. Results

### 3.1. Study Selection

**Figure 1:**
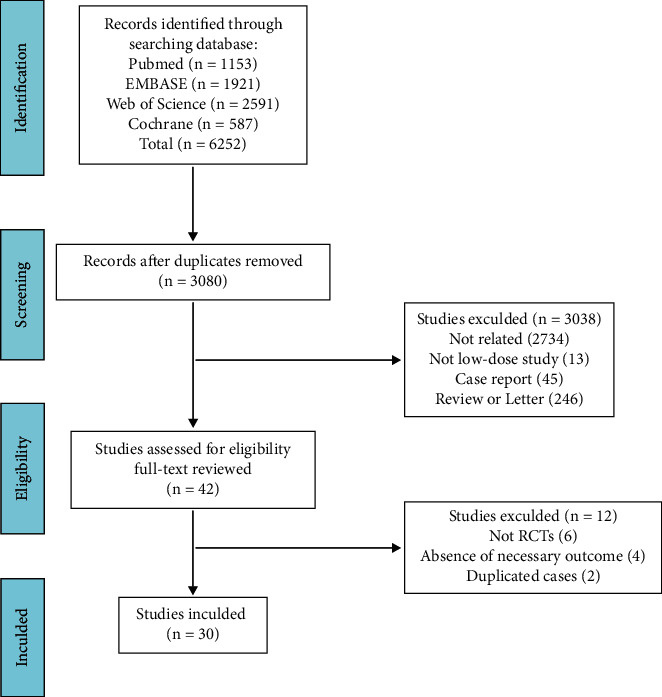
Flow diagram depicting the literature review, search strategy, and selection process.

The systematic search yielded 6,252 articles, of which 3172 were duplicates. 3,038 studies were excluded by screening the title and abstract, and 12 studies were reasonably considered improper after full-text reviewing. Eventually, 30 studies were finally included in this meta-analysis (Figure 1) [24–53].

### 3.2. Characteristics of Included Studies

A total of 30 randomized controlled trials comprising 1,865 patients undergoing elective spine surgery were included. Of the patients, 1,006 cases were treated with perioperative low-dose ketamine, and 859 cases were administrated with placebo or basic analgesic alone. The characteristics of the included studies were demonstrated in Table 1. The baseline characteristics of the two groups were matched.

### 3.3. Quality Assessment of the Selected Studies

**Figure 2:**
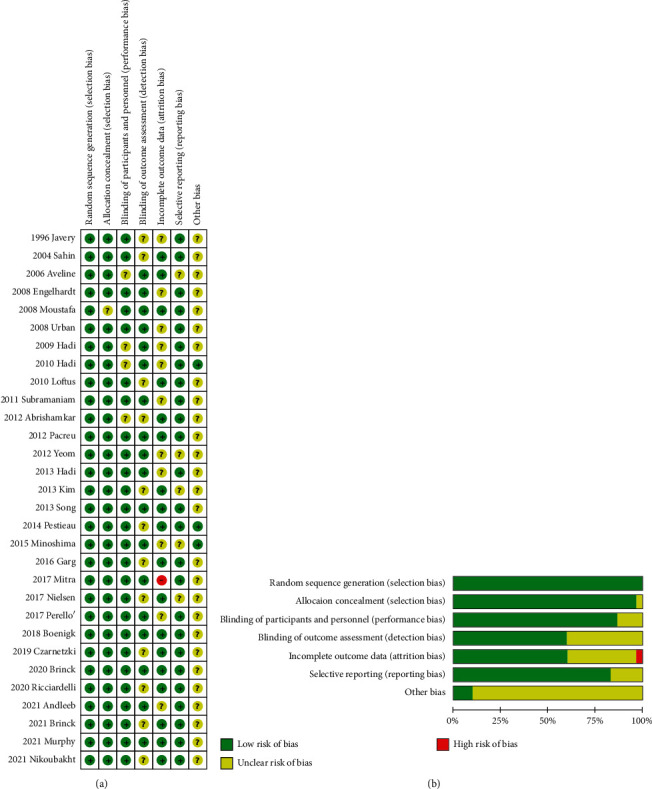
Risk of bias. (a) A summary table of each risk of bias item for each study; (b) a plot of the distribution of each risk of bias item. “+”: low risk of bias; “?”: unclear risk of bias; “–”: high risk of bias.

The majority of the studies had a “low risk” or an “unclear risk” assessment according to the Cochrane Handbook. The pooled risks of bias is presented in Figures 2(a) and 2(b).

### 3.4. Postoperative Pain Intensity

#### 3.4.1. Pain Intensity at Rest at 6 h Postoperatively

**Figure 3:**
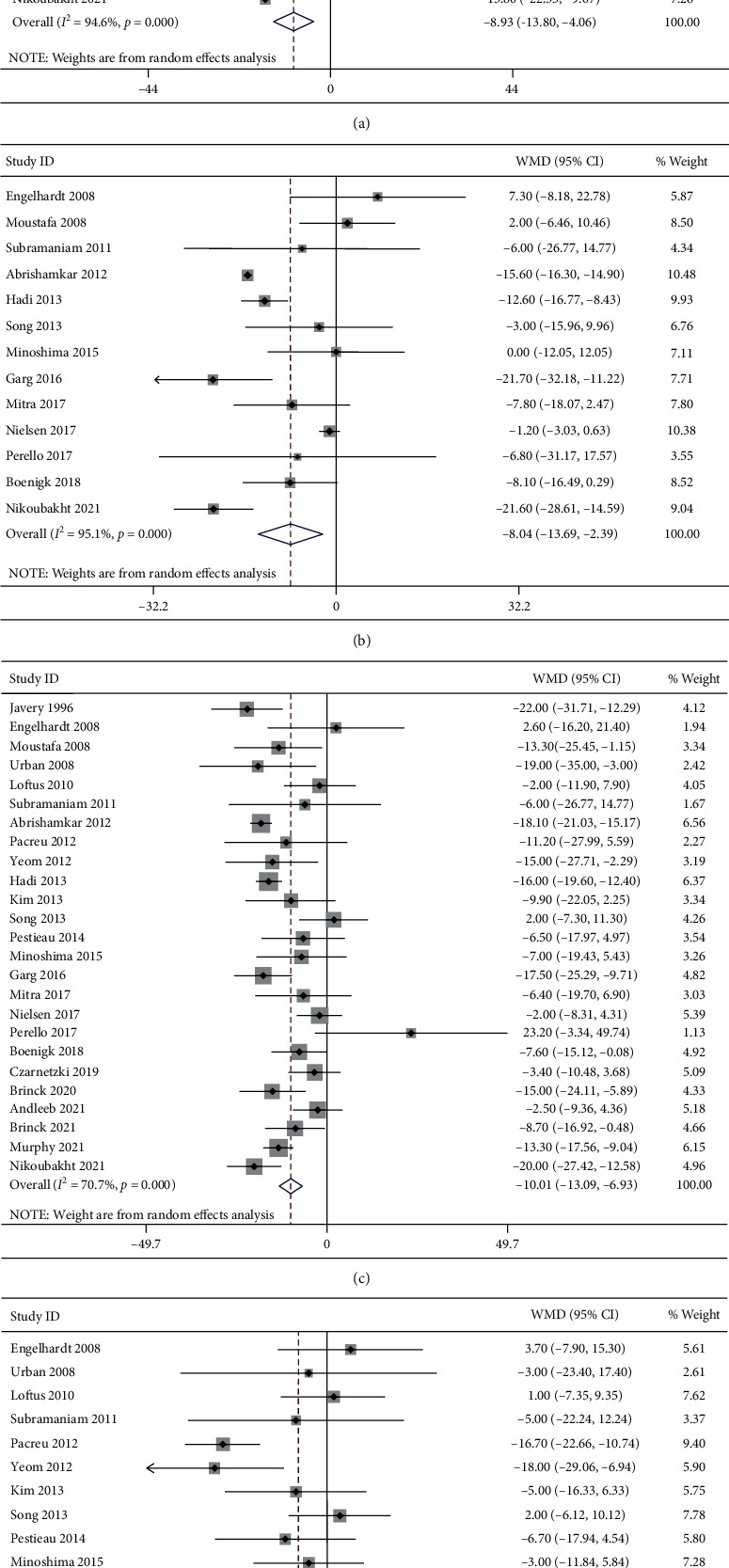
Forest plot of the pain intensity at rest for the ketamine group and control group. (a) 6 h; (b) 12 h; (c) 24 h; (d) 48 h.

A total of 16 studies reported the pain intensity at rest at 6 h postoperatively. Significant heterogeneity was detected (*I*^2^ = 94.6%, *P* < 0.001). The 16 studies included 587 patients in the ketamine group and 457 patients in the control group. The pooled results revealed significantly lower pain scores at rest at 6 h postoperatively in the ketamine than the control group (WMD, −8.93; 95% CI −13.80 to −4.06, *P* < 0.001, GRADE = moderate) (Figure 3(a)).

#### 3.4.2. Pain Intensity at Rest at 12 h Postoperatively

A total of 13 studies reported the pain intensity at rest at 12 h postoperatively. Significant heterogeneity was detected (*I*^2^ = 95.1%, *P* < 0.001). The 14 studies included 350 patients in the ketamine group and 353 patients in the control group. The pooled results revealed significantly lower pain scores at rest at 12 h postoperatively in the ketamine than the control group (WMD, −8.04; 95% CI −13.69 to −2.39, *P*=0.005, GRADE = moderate) (Figure 3(b)).

#### 3.4.3. Pain Intensity at Rest at 24 h Postoperatively

A total of 25 studies reported the pain intensity at rest at 24 h postoperatively. Significant heterogeneity was detected (*I*^2^ = 70.7%, *P* < 0.001). The 25 studies included 907 patients in the ketamine group and 759 patients in the control group. The pooled results revealed significantly lower pain scores at rest at 24 h postoperatively in the ketamine than the control group (WMD, −10.01; 95% CI −13.09 to −6.93, *P* < 0.001, GRADE = moderate) (Figure 3(c)).

#### 3.4.4. Pain Intensity at Rest at 48 h Postoperatively

A total of 16 studies reported the pain intensity at rest at 48 h postoperatively. Significant heterogeneity was detected (*I*^2^ = 56.1%, *P*=0.003). The 16 studies included 565 patients in the ketamine group and 474 patients in the control group. The pooled results revealed significantly lower pain scores at rest at 48 h postoperatively in the ketamine than the control group (WMD, −4.63; 95% CI −8.34 to −0.92, *P*=0.014, GRADE = moderate) (Figure 3(d)).

#### 3.4.5. Pain Intensity during Mobilization at 6 h Postoperatively

**Figure 4:**
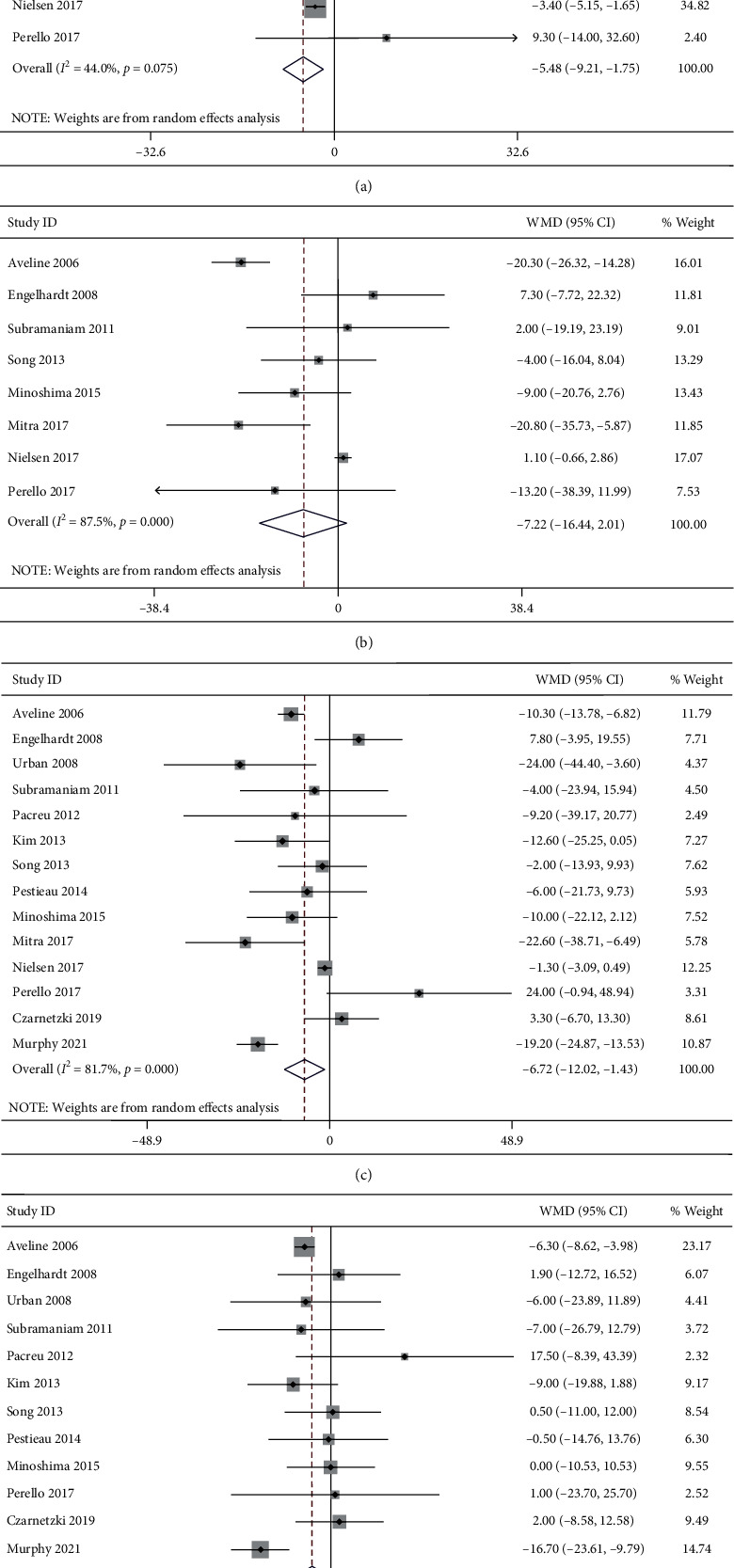
Forest plot of the pain intensity during mobilization for the ketamine group and control group. (a) 6 h; (b) 12 h; (c) 24 h; (d) 48 h.

A total of 9 studies reported the pain intensity during mobilization at 6 h postoperatively. Significant heterogeneity was detected (*I*^2^ = 44.0%, *P*=0.075). The 9 studies included 239 patients in the ketamine group and 227 patients in the control group. The pooled results revealed significantly lower pain scores during mobilization at 6 h postoperatively in the ketamine than the control group (WMD, −5.48; 95% CI −9.21 to −1.75, *P*=0.004, GRADE = low) (Figure 4(a)).

#### 3.4.6. Pain Intensity during Mobilization at 12 h Postoperatively

A total of 8 studies reported the pain intensity during mobilization at 12 h postoperatively. Significant heterogeneity was detected (I^2^ = 87.5%, *P* < 0.001). The 8 studies included 204 patients in the ketamine group and 210 patients in the control group. The pooled results revealed no significant difference in pain scores during mobilization at 12 h postoperatively between groups (WMD, −7.22; 95% CI −16.44 to 2.01, *P*=0.125, GRADE = low) (Figure 4(b)).

#### 3.4.7. Pain Intensity during Mobilization at 24 h Postoperatively

A total of 14 studies reported the pain intensity during mobilization at 24 h postoperatively. Significant heterogeneity was detected (I^2^ = 81.7%, *P* < 0.001). The 14 studies included 425 patients in the ketamine group and 402 patients in the control group. The pooled results revealed significantly lower pain scores during mobilization at 24 h postoperatively in the ketamine than the control group (WMD, −6.72; 95% CI −12.02 to −1.43, *P* < 0.001, GRADE = moderate) (Figure 4(c)).

#### 3.4.8. Pain Intensity during Mobilization at 48 h Postoperatively

A total of 12 studies reported the pain intensity during mobilization at 48 h postoperatively. Significant heterogeneity was detected (*I*^2^ = 43.3%, *P* < 0.054). The 12 studies included 338 patients in the ketamine group and 316 patients in the control group. The pooled results revealed significantly lower pain scores during mobilization at 48 h postoperatively in the ketamine than the control group (WMD, −4.52; 95% CI −8.66 to −0.38, *P*=0.032, GRADE = moderate) (Figure 4(d)).

#### 3.4.9. Subgroup Analysis by Ages

The heterogeneity of pain scores was significantly decreased after categorizing the studies into pediatric studies and adult studies. For adult patients, the pooled results revealed that the pain scores at rest at 6 h (WMD, −11.73; 95% CI −17.09 to −6.38, *P* < 0.001; GRADE = moderate), 12 h (WMD, −11.21; 95% CI −17.64 to −4.78, *P*=0.001, GRADE = moderate), 24 h (WMD, −10.86; 95% CI −14.11 to −7.62, *P* < 0.001, GRADE = moderate), 48 h (WMD, −5.37; 95% CI −10.23 to −0.50, *P*=0.031, GRADE = moderate) and during mobilization at 6 h (WMD, −6.28; 95% CI −10.41 to −2.15, *P*=0.003, GRADE = moderate), 24 h (WMD, −9.28; 95% CI −15.40 to −3.17, *P*=0.003, GRADE = moderate), and 48 h (WMD, −5.88; 95% CI −11.13 to −0.64, *P*=0.028, GRADE = moderate) postoperatively were significantly lower in the ketamine group than the control group. However, for pediatric patients, there were no significant difference in pain scores at rest at 6 h (WMD, 1.05; 95% CI −4.47 to 6.57, *P*=0.708, GRADE = moderate), 12 h (WMD, 1.76; 95% CI −4.36 to 7.88, *P*=0.573, GRADE = moderate), 24 h (WMD, −4.36; 95% CI −13.04 to 4.31, *P*=0.324, GRADE = moderate), 48 h (WMD, −3.09; 95% CI −8.03 to 1.86, *P*=0.222, GRADE = moderate) and during mobilization at 6 h (WMD, −0.34; 95% CI −10.78 to 10.11, *P*=0.950, GRADE = moderate), 12 h (WMD, −3.87; 95% CI −15.99 to 8.24, *P*=0.531), 24 h (WMD, 1.67; 95% CI −10.97 to 14.31, *P*=0.796, GRADE = moderate), and 48 h (WMD, 0.40; 95% CI −6.63 to 7.42, *P*=0.912, GRADE = moderate) postoperatively between groups.

#### 3.4.10. Subgroup Analysis by Administration Times

The heterogeneity of pain scores was significantly decreased after categorizing the studies into intrasubgroup, postsubgroup, and intrasubgroup + postsubgroup, according to the administration times of ketamine.

At rest, the pain scores at 6 h (WMD, −10.72; 95% CI −15.35 to −6.09, *P* < 0.001; WMD, -17.90, GRADE = moderate; 95% CI −26.23 to −9.58, *P* < 0.001, GRADE = moderate), 12 h (WMD, −13.00; 95% CI −20.44 to −5.55, *P*=0.001, GRADE = moderate; WMD, −14.90; 95% CI −20.34 to −9.46, *P* < 0.001, GRADE = low), and 24 h (WMD, −11.51; 95% CI −15.93 to −7.08, *P* < 0.001, GRADE = moderate; WMD, −14.90; 95% CI −19.97 to −9.84, *P* < 0.001, GRADE = high) after surgery were significantly lower in the ketamine group when the drugs were administrated intraoperatively and postoperatively or solely postoperatively. However, when ketamine was administrated solely intraoperatively, the only significant difference in pain scores was detected at 24 h postoperatively (WMD, −5.01; 95% CI −9.82 to −0.19, *P*=0.042, GRADE = moderate) and no significant difference at 6 h (WMD, −5.81; 95% CI −12.39 to 0.77, *P*=0.084, GRADE = low) and 12 h (WMD, −2.49; 95% CI −7.61 to 2.64, *P*=0.342, GRADE = low). As there was only one study in postsubgroup that reported the data at 48 h postoperatively, we excluded this subgroup for analysis at this terminated point. The pain scores at 48 h postoperatively were significantly lower in the ketamine group when the drugs were administrated intraoperatively and postoperatively (WMD, −6.71; 95% CI −12.39 to −1.04, *P*=0.020, GRADE = moderate) while no significant difference in intrasubgroup (WMD, 2.42; 95% CI −4.28 to 9.13, *P*=0.479, GRADE = low).

During mobilization, there was no data reported by studies in postsubgroup. Thus, only intrasubgroup + postsubgroup and intrasubgroup were included for analysis. The pain scores at 6 h after surgery were significantly lower in the ketamine group when the drugs were administrated intraoperatively (WMD, −7.28; 95% CI −13.29 to −1.28, *P*=0.017, GRADE = moderate), and the pain scores at 24 h after surgery were significantly lower in the ketamine group when the drugs were administrated intraoperatively and postoperatively (WMD, −9.43; 95% CI −18.35 to −0.51, *P*=0.038, GRADE = moderate). However, there were no significant difference in pain scores at 6 h (WMD, −4.34; 95% CI −11.80 to 3.11, *P*=0.253, GRADE = moderate) and 12 h between groups in intrasubgroup + postsubgroup (WMD, −2.54; 95% CI −13.00 to 7.93, *P*=0.635, GRADE = low), and there were no significant difference in pain scores at 12 h (WMD, −12.68; 95% CI −30.18 to 4.82, *P*=0.156, GRADE = low) and 24 h (WMD, −8.62; 95% CI −17.28 to 0.05, *P*=0.051, GRADE = moderate) between groups in intrasubgroup. We were unable to perform subgroup analysis for pain scores at 48 h after surgery because there were only one study in intrasubgroup.

### 3.5. Postoperative Opioid Consumption

#### 3.5.1. Cumulative Opioid Consumption in the First 12 h Postoperatively

**Figure 5:**
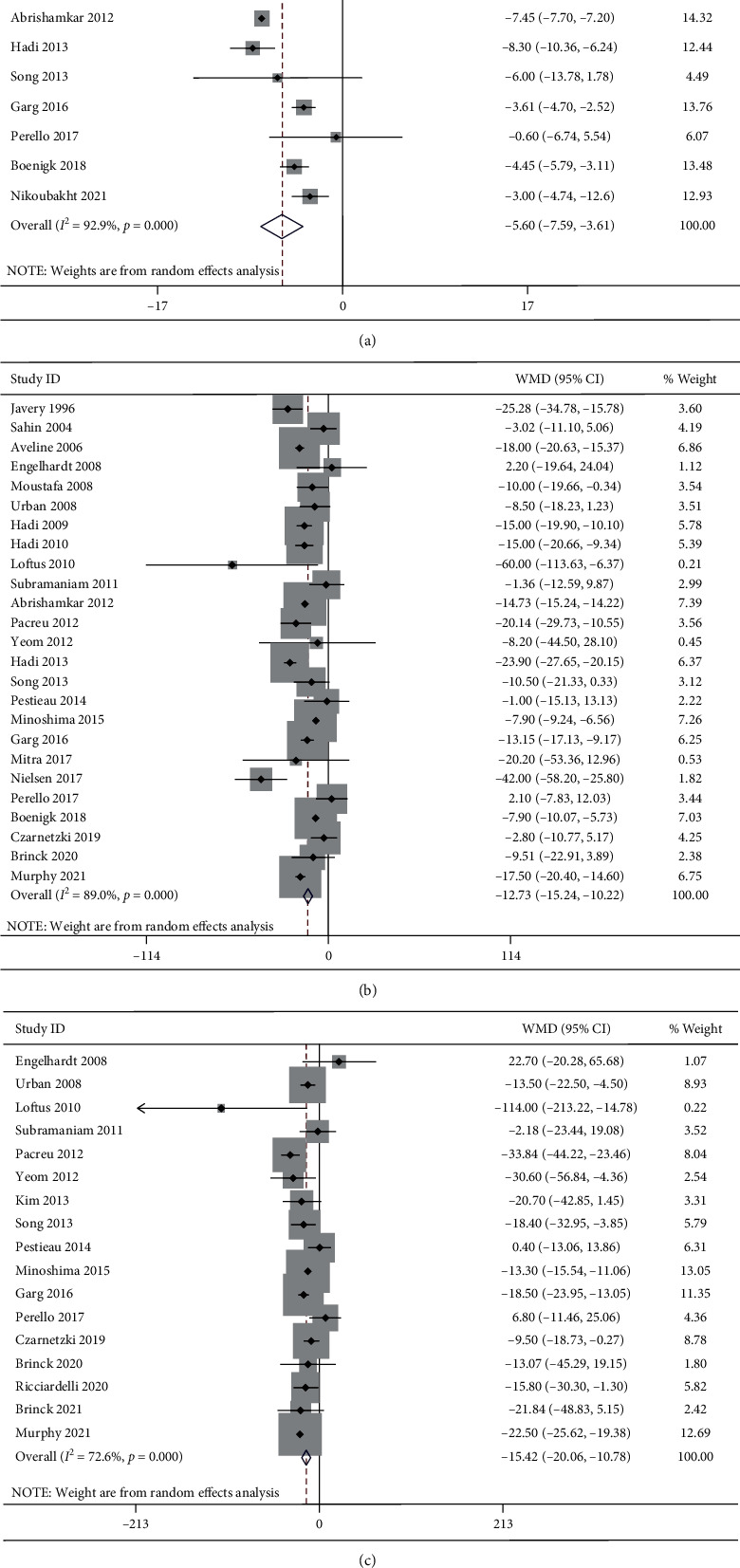
Forest plot of the cumulative opioid consumption for the ketamine group and control group. (a) First 12 h; (b) first 24 h; (c) first 48 h.

A total of 10 studies reported the cumulative opioid consumption in the first 12 h postoperatively. Significant heterogeneity was detected (*I*^2^ = 92.9%, *P* < 0.001). The 10 studies included 252 patients in the ketamine group and 254 patients in the control group. The pooled results revealed significantly reduced cumulative morphine equivalent in the first 12 h postoperatively in the ketamine than the control group (WMD, −5.60; 95% CI −7.59 to −3.61, *P* < 0.001, GRADE = moderate) (Figure 5(a)).

#### 3.5.2. Cumulative Opioid Consumption in the First 24 h Postoperatively

A total of 25 studies reported the cumulative opioid consumption in the first 24 h postoperatively. Significant heterogeneity was detected (I^2^ = 89.0%, *P* < 0.001). The 25 studies included 759 patients in the ketamine group and 692 patients in the control group. The pooled results revealed significantly reduced cumulative morphine equivalent in the first 24 h postoperatively in the ketamine than the control group (WMD, −12.73; 95% CI −15.24 to −10.22, *P* < 0.001, GRADE = moderate) (Figure 5(b)).

#### 3.5.3. Cumulative Opioid Consumption in the First 48 h Postoperatively

A total of 17 studies reported the cumulative opioid consumption in the first 48 h postoperatively. Significant heterogeneity was detected (*I*^2^ = 72.6%, *P* < 0.001). The 17 studies included 645 patients in the ketamine group and 505 patients in the control group. The pooled results revealed significantly reduced cumulative morphine equivalent in the first 48 h postoperatively in the ketamine than the control group (WMD, −15.42; 95% CI −20.06 to −10.78, *P* < 0.001, GRADE = moderate) (Figure 5(c)).

#### 3.5.4. Subgroup Analysis by Ages

The heterogeneity of cumulative opioid consumption was significantly decreased after categorizing the studies into pediatric studies and adult studies. For adult patients, the pooled results revealed that the cumulative opioid consumption in the first 12 h (WMD, −5.95; 95% CI −8.02 to −3.88, *P* < 0.001, GRADE = moderate), 24 h (WMD, −14.42; 95% CI −16.99 to −11.85, *P* < 0.001, GRADE = high), and 48 h (WMD, −19.24; 95% CI −24.16 to −14.32, *P* < 0.001, GRADE = high) postoperatively was significantly reduced in the ketamine group than the control group. For pediatric patients, cumulative opioid consumption in the first 24 h (WMD, −5.80; 95% CI −10.17 to −1.42, *P*=0.009, GRADE = moderate) was significantly reduced in the ketamine group than the control group while no significant difference in the first 12 h (WMD, −1.42; 95% CI −6.91 to 4.08, *P*=0.613, GRADE = low) and 48 h (WMD, −5.82; 95% CI −15.75 to 4.12, *P*=0.251, GRADE = moderate) postoperatively between groups.

#### 3.5.5. Subgroup Analysis by Administration Times:

The heterogeneity of cumulative opioid consumption was significantly decreased after categorizing the studies into intrasubgroup, postsubgroup, and intrasubgroup + postsubgroup according to the administration times of ketamine. The cumulative opioid consumption in the first 12 h (WMD, −4.48; 95% CI −8.35 to −0.61, *P*=0.023, GRADE = moderate; WMD, −5.21; 95% CI −8.02 to −2.40, *P* < 0.001, GRADE = moderate), 24 h (WMD, -12.91; 95% CI -18.85 to -6.97, *P* < 0.001, GRADE = high; WMD, −13.41; 95% CI −17.87 to −8.95, *P* < 0.001, GRADE = moderate), and 48 h (WMD, −19.05; 95% CI −25.49 to −12.62, *P* < 0.001, GRADE = moderate; WMD, −18.63; 95% CI −23.98 to −13.28, *P* < 0.001, GRADE = low) after surgery was significantly reduced in the ketamine group when the drugs were administrated intraoperatively and postoperatively or solely postoperatively. However, for intrasubgroup, only the cumulative opioid consumption in the first 24 h was significantly reduced in the ketamine group (WMD, −16.74; 95% CI −22.73 to −10.75, *P* < 0.001, GRADE = moderate). As there was only one study in intrasubgroup that reported the data in the first 12 h postoperatively, we excluded this subgroup for analysis at this terminated point.

### 3.6. Time to First Request for Analgesic after Surgery

**Figure 6:**
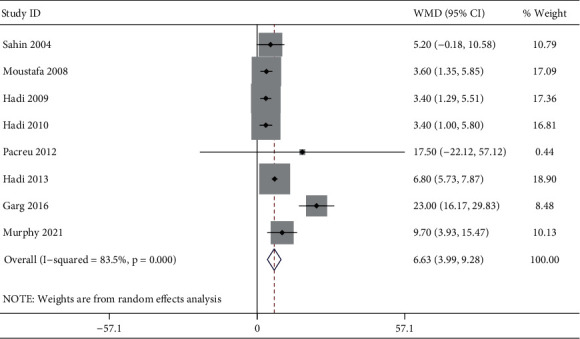
Forest plot of the time to first request for analgesic after surgery for the ketamine group and control group.

A total of 8 studies reported the time to first request for analgesic after surgery. Significant heterogeneity was detected (*I*^2^ = 83.5%, *P* < 0.001). The 8 studies included 196 patients in the ketamine group and 174 patients in the control group. The pooled results revealed significantly prolonged time to first request for analgesic after surgery in the ketamine than the control group (WMD, 6.63; 95% CI 3.99 to 9.28, *P* < 0.001, GRADE = moderate) (Figure 6).

### 3.7. Adverse Events with the Administration of Ketamine

#### 3.7.1. Central Nervous System Adverse Events

**Figure 7:**
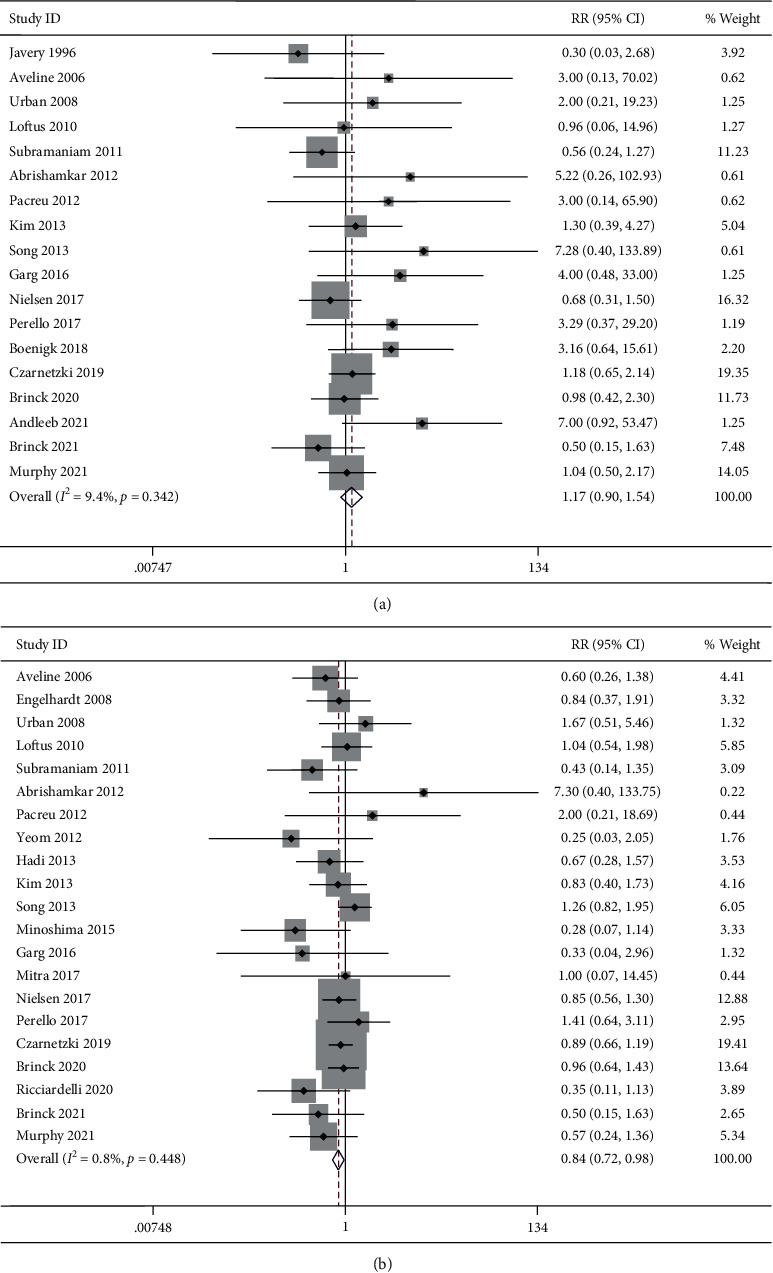
Forest plot of the incidence of adverse events for the ketamine group and control group. (a) CNS adverse events; (b) PONV.

CNS adverse events including hallucination, confusion, disorientation, visual disturbance, sedation, nightmare, and drowsiness were reported by 18 studies. No substantial heterogeneity was detected (*I*^2^ = 9.4%, *P*=0.342). The incidence of CNS adverse event was 13.7% (103/752) in the ketamine group and 11.6% (72/623) in the control group. The pooled results revealed no significant difference in the incidence of CNS adverse event between groups (RR, 1.17; 95% CI 0.90 to 1.54, *P*=0.243, GRADE = moderate) (Figure 7(a)).

#### 3.7.2. Postoperative Nausea and Vomiting

PONV were reported by 21 studies. No substantial heterogeneity was detected (*I*^2^ = 0.8%, *P*=0.448). The incidence of PONV adverse event was 27.8% (215/772) in the ketamine group and 33.9% (213/629) in the control group. The pooled results revealed a significantly lower incidence of PONV in the ketamine group than the control group (RR, 0.84; 95% CI 0.72 to 0.98, *P*=0.028, GRADE = moderate) (Figure 7(b)).

#### 3.7.3. Subgroup Analysis by Ages

We were unable to perform subgroup analysis for CNS adverse events because there was only one study in pediatric subgroup. The pooled results revealed no significant difference in the incidence of PONV between groups for both adult (RR, 0.87; 95% CI 0.74 to 1.02, *P*=0.083, GRADE = moderate) and pediatric subgroup (RR, 0.68; 95% CI 0.43 to 1.09, *P*=0.113, GRADE = low).

#### 3.7.4. Subgroup Analysis by Administration Times

The pooled results revealed no significant difference in the incidence of CNS adverse events between groups for intrasubgroup (RR, 1.10; 95% CI 0.66 to 1.84, *P*=0.705, GRADE = moderate), postsubgroup (RR, 1.30; 95% CI 0.65 to 2.57, *P*=0.455, GRADE = moderate), and intrasubgroup + postsubgroup (RR, 1.13; 95% CI 0.79 to 1.63, *P*=0.504, GRADE = moderate). Similarly, the pooled results revealed no significant difference in the incidence of PONV between groups for intrasubgroup (RR, 0.89; 95% CI 0.69 to 1.15, *P*=0.365, GRADE = moderate), postsubgroup (RR, 0.80; 95% CI 0.33 to 1.90, *P*=0.611, GRADE = low), and intrasubgroup + postsubgroup (RR, 0.86; 95% CI 0.69 to 1.06, *P*=0.150, GRADE = moderate).

## 4. Discussion

In several previous reviews, the administration of perioperative low-dose ketamine has demonstrated an opioid-sparing effect in patients undergoing major surgery and could mitigate opioid-induced hyperalgesia and acute opioid tolerance shown to occur in these patients [2, 54, 55]. However, these literature focused on a broad range of surgical procedures, whether this hold true for spine surgery remained controversial. Although a meta-analysis by Pendi et al. has reported that supplemental perioperative ketamine was effective in pain management following spine surgery, this study did not consider the impact of patient ages and administration time of drugs [56]. As previous studies have reported an insignificant reduction in pain score and opioid consumption for pediatric patients undergoing perioperative low-dose ketamine, and only intraoperative administration could not prolong the analgesia time in adult surgery, the results by Pendi et al. may be skewed by the heterogeneous of included studies [3, 57]. In the current study, we additionally performed subgroup analysis according to ages and administration time in order to report the efficacy of perioperative low-dose ketamine more precisely.

### 4.1. Postoperative Pain Intensity and Cumulative Opioid Consumption

Patients administrated low-dose ketamine reported significantly less pain and corresponding decreased need for opioids up to 48 h postoperatively in the overall analysis, which was consistent with previous studies [20]. Inconsistent with our results, a recent meta-analysis focused on spine surgery by Pendi et al. pooled the data of pediatric and adult and reported that the analgesic effect of ketamine supplementation may be only limited to the first 24 h after surgery [56]. However, the age-related differences in pharmacokinetic could impact the reliability of their results [45]. In the current study, an analgesic and morphine-sparing effect was only detected in adult patients. For pediatric patients, the low-dose ketamine failed to decrease the pain intensity and only reduced the cumulative opioid consumption in the first 24 h postoperatively. This finding was in concert with the meta-analysis by Dahmani et al., who indicated that perioperative administration of ketamine in children could not change early postoperative pain and analgesic use [58]. Pharmacokinetic studies had suggested a shorter context-sensitive half-time and a higher requirement of ketamine doses to maintain the steady-state concentrations in pediatric populations compared to adults [59, 60]. Therefore, the low-dose ketamine used in pediatric patients was not as sufficient as in adults to suppress the NMDA receptor pathway [40].

Subgroup analysis also indicated that intraoperative administration of ketamine solely was not as effective as postoperative or intraoperative and postoperative administration to prolong the analgesia time and reduced opioid consumptions, especially during 24 h to 48 h after surgery. Central sensitization was associated with hyperalgesia and opioid tolerance [44, 45]. Repetitive and high frequency noxious stimulus from C-fibers via activation of NMDA receptor could evoke this process not only during surgery but also in the postoperative period [41]. Therefore, the withdrawal hyperalgesia and acute opioid tolerance may continue and even develop long after surgery [61]. Our finding was consistent with the results of a previous meta-analysis by Wang et al., who reported that the analgesic effect and morphine-sparing effect provided by intraoperative administration of ketamine solely was very limited for adult surgery, although in the first 24 h postoperatively [62]. Thus, to obtain a beneficial effect in postoperative pain management, low-dose ketamine administrated in postoperative period or through the perioperative period may be the better choice.

### 4.2. Adverse Events with the Administration of Ketamine

A common concern with the use of ketamine has been its side effects including CNS adverse events and PONV [63]. Consistent with a previous large meta-analysis of ketamine adjuncts to opioid for pain control which included 130 RCTs of 4,588 participants, our results indicated that the incidence of ketamine-related adverse events has not been increased compared to those who only received opioids, in both pediatric and adult patients [20].

The meta-analysis by Wang et al. reported that the rate of psychotomimetic events was significantly higher in patients administrated low-dose ketamine intraoperatively and postoperatively [62]. However, Wang et al. pooled the data of various surgical procedures in adult patients, including hemorrhoidectomy, radical prostatectomy, laparoscopic cholecystectomy, thoracotomy, and lumbar fusion, which was highly heterogeneous and may skew the results. When solely focused on spine surgery, this study revealed that postoperative or intraoperative and postoperative administration of low-dose ketamine would not increase the risk of adverse events, in addition to its prolonged analgesic effect and morphine-sparing effect.

### 4.3. Limitations

We believe that this meta-analysis has presented valuable results for many anesthesiologists and spinal surgeons, although with some limitations. Firstly, although we performed subgroup analysis, there was still significant heterogeneity in most of the analyses, which might be due to different study designs, modes of delivery, dosages, and procedures. Secondly, combining multiple treatment arms may have produced a moderating effect. Thirdly, chronic opioid-dependent could magnitude the analgesic effect of ketamine; however, some studies did not clarify the usage of preoperative opioid, leaving it unclear whether opioid-tolerant patients were included [44]. Also, although this study indicated that low-dose ketamine could decrease the postoperative pain intensity and opioid use, the optimal protocol, including mode of administration, dosage, and timing, were not obtained. Later, although the participants were categorized into pediatric (10 to 17 years of age) and adult (≥18 years of age) in this study, the adult participants could not be further categorized by middle and old age due to the design of included RCTs. According to the mean age of each study, there was only one study that fulfilled the definition of old age participants (≥65 years of age) [64–66]. Considering that the old age people are more susceptible to spine disorders, further studies should focus on this population, who are the main surgical candidates. Last, although we have applied the Egger's test to assess the publication bias, the potential language bias is inevitable because clinical investigators working in non-English-speaking countries are more likely to publish their positive findings in an international English-language journal while reporting negative results in their local languages [67].

## 5. Conclusion

Perioperative low-dose ketamine demonstrated analgesic and morphine-sparing effect with no increased adverse events after spine surgery. However, the effect was not significant in pediatric patients. Only postoperative or intraoperative and postoperative administration could prolong the analgesic time up to 48 h postoperatively. Further studies should focus on the optimal protocol of ketamine administration and its effect on old age participants.

## Figures and Tables

**Table 1:** Characteristics of the included studies.

Author	Year	Group	Sample size	Age (years)			Sex (M/F)	Weight (kg)			Mode	Bolus (mg kg^−1^)	Infusion (*μ*g kg^−1^ min^−1^)	Dose in IV-PCA(mg ml^−1^)	Timing	Postoperative Opioid	Control
Javery	1996	Ketamine	22	37.3	±	9.9	18/4	78.2	±	13.1	IV-PCA			1.0	Postoperative	Morphine	Basic
		Control	20	39.5	±	7.2	18/2	83.9	±	10.7							

Sahin	2004	Ketamine	17	46.5	±	7.3	9/8	80.4	±	16.1	B	0.5			Intraoperative	Morphine	Saline
		Control	14	46.1	±	13.3	8/6	78.7	±	13.2							

Aveline	2006	Ketamine	23	48.3	±	12.3	11/12	74.2	±	6.3	B	0.15			Intraoperative	Morphine	Basic
		Control	23	44.4	±	11.2	10/13	68.4	±	12.8							

Engelhardt	2008	Ketamine	16	14.8	±	1.7	2/14	59.3	±	16.5	B+ivgtt	0.5	4.0		Intraoperative	Morphine	Saline
		Control	18	15.5	±	1.2	3/15	56.5	±	10.4							

Moustafa	2008	Ketamine	16	13.1	±	5.08	7/9	39.7	±	8.63	ivgtt		1.0		Intraoperative	Morphine	Basic
		Control	16	12.8	±	6.07	6/10	34.6	±	9.68							

Urban	2008	Ketamine	12	53	±	12		80	±	19	B+ivgtt	0.2	0.03		Intraoperative Postoperative	Hydromorphone	Saline
		Control	12	48	±	9		78	±	19							

Hadi	2009	Ketamine	20	22	±	2	7/13	55	±	15	ivgtt		1.0		Intraoperative	Morphine	Basic
		Control	20	21	±	2	8/12	54	±	13							

Hadi	2010	Ketamine	15	56.1	±	10.5	7/8	66	±	13	ivgtt		1.0		Intraoperative	Morphine	Saline
		Control	15	53.5	±	10.2	3/12	68	±	12							

Loftus	2010	Ketamine	52	51.7	±	14.2	33/19	95.4	±	17.7	B+ivgtt	0.5	10.0		Intraoperative	Morphine	Saline
		Control	50	51.4	±	14.4	28/22	89.3	±	23.8							

Subramaniam	2011	Ketamine	15	57.2	±	12.2	8/7	86.2	±	24.4	B+ivgtt	0.15	2.0		Intraoperative Postoperative	Hydromorphone	Saline
		Control	15	56.5	±	13.6	7/8	81.4	±	19.2							

Abrishamkar	2012	Ketamine	22	49	±	1.32	5/18				ivgtt		8.33		Postoperative	Morphine	Basic
		Control	23	45	±	1.64	10/12										

Pacreu	2012	Ketamine	10	52.9	±	12.6	3/7	69.5	±	7.2	B+ivgtt+IV-PCA	0.5	2.5	0.5	Intraoperative Postoperative	Methadone	Saline
		Control	10	61.3	±	11.7	3/7	75.9	±	9.8							

Yeom	2012	Ketamine	20	61.0	±	10.0	5/15	59.1	±	13.5	B+ivgtt	0.2	0.5		Intraoperative Postoperative	Fentanyl	Saline
		Control	20	64.5	±	11.5	7/13	64.8	±	10.6							

Hadi	2013	Ketamine	30	55	±	2.5	13/17	70	±	2.5	ivgtt		1.0		Intraoperative Postoperative	Morphine	Saline
		Control	15	51	±	2.5	8/7	71	±	2.6							

Kim	2013	Ketamine	35	56	±	9.5	15/20	63.4	±	10.5	B+ivgtt	0.5	1.0-2.0		Intraoperative Postoperative	Fentanyl	Saline
		Control	17	56	±	13	9/8	66	±	13							

Song	2013	Ketamine	24	52.3	±	8.8	0/24	59	±	7	B+ivgtt	0.3	0.04		Intraoperative Postoperative	Fentanyl	Saline
		Control	25	53.8	±	7.8	0/25	60	±	10							

Pestieau	2014	Ketamine	29	14.3	±	1.8	5/24	60	±	18	B+ivgtt	0.5	0.1-0.2		Intraoperative Postoperative	Morphine	Saline
		Control	21	14.5	±	1.5	7/14	59	±	14							

Minoshima	2015	Ketamine	17	15	±	2	1/16	45	±	6	B+ivgtt	0.5	2.0		Intraoperative Postoperative	Morphine	Saline
		Control	19	14	±	2	0/19	46	±	4							

Garg	2016	Ketamine	22	36.5	±	13.4	13/9	61.5	±	14.6	B+ivgtt	0.25	4.2		Postoperative	Morphine	Saline
		Control	22	36.3	±	14.3	16/6	65.3	±	15.5							

Mitra	2017	Ketamine	14	33.9	±	17.2	7/7	56.4	±	10.1	B+ivgtt	0.5	4.2		Intraoperative	Fentanyl	Saline
		Control	14	33.5	±	15.2	6/8	57.9	±	11.3							

Nielsen	2017	Ketamine	74	57	±	14	28/46	77	±	14	B+ivgtt	0.5	4.2		Intraoperative	Morphine	Saline
		Control	73	55	±	13	21/52	78	±	18							

Perello ´	2017	Ketamine	21	14.3	±	1.9	5/16	54.3	±	10.8	B+ivgtt	0.5	2.0		Intraoperative Postoperative	Morphine	Saline
		Control	23	14.3	±	1.8	6/17	57.6	±	12.6							

Boenigk	2018	Ketamine	49	54.5	±	13.4	24/25	78.5	±	7.2	B+ivgtt	0.2	2		Postoperative	Hydromorphone	Saline
		Control	62	55.7	±	13.2	29/33	85.4	±	11.5							

Czarnetzki	2019	Ketamine	80	65.8	±	13.8	39/41	79.8	±	4.3	B+ivgtt	0.25	1.67-4.17		Intraoperative Postoperative	Morphine	Saline
		Control	80	65.6	±	12.6	42/38	79.7	±	3.4							

Brinck	2020	Ketamine	127	54.2	±	13.2	40/87	76	±	14	B+ivgtt	0.5	2-10		Intraoperative	Oxycodone	Saline
		Control	62	55.5	±	12	26/36	79	±	15							

Ricciardelli	2020	Ketamine	24	13.4	±	1.7	2/22	60.9	±	17.4	B+ivgtt	0.5	3.33		Intraoperative Postoperative	Morphine	Saline
		Control	25	14.7	±	2.2	8/17	61.6	±	14.2							

Andleeb	2021	Ketamine	30	43.8	±	14.6	15/15	57.4	±	11.4	ivgtt		8.33		Intraoperative	Morphine	Saline
		Control	30	39.8	±	14.1	14/16	58	±	12.7							

Brinck	2021	Ketamine	75	60	±	13.5	41/34	77.1	±	14.5	IV-PCA			0.25-0.75	Postoperative	Oxycodone	Saline
		Control	25	56	±	11	14/11	77.7	±	15.6							

Murphy	2021	Ketamine	66	59.3	±	16.4	32/34	64	±	11	ivgtt		1.67-5.00		Intraoperative Postoperative	Hydromorphone	Saline
		Control	61	65.3	±	10.6	27/34	62	±	11							

Nikoubakht	2021	Ketamine	29	52.8	±	12.2	17/12				ivgtt		1.67		Intraoperative	Morphine	Saline
		Control	29	53.8	±	13.9	18/11										

B indicates intravenous bolus; ivgtt, intravenously guttae; IV-PCA, intravenous patient-controlled analgesia.

## Data Availability

The data used to support the findings of this study are available from the corresponding author upon request.

## Supplementary Materials

Supplementary Materials Supplementary Figure S1: subgroup analysis of pain intensity at rest at 6 h postoperatively by ages. Supplementary Figure S2: subgroup analysis of pain intensity at rest at 12 h postoperatively by ages. Supplementary Figure S3: subgroup analysis of pain intensity at rest at 24 h postoperatively by ages. Supplementary Figure S4: subgroup analysis of pain intensity at rest at 48 h postoperatively by ages. Supplementary Figure S5: subgroup analysis of pain intensity during mobilization at 6 h postoperatively by ages. Supplementary Figure S6: subgroup analysis of pain intensity during mobilization at 12 h postoperatively by ages. Supplementary Figure S7: subgroup analysis of pain intensity during mobilization at 24 h postoperatively by ages. Supplementary Figure S8: subgroup analysis of pain intensity during mobilization at 48 h postoperatively by ages. Supplementary Figure S9: subgroup analysis of pain intensity at rest at 6 h postoperatively by administration times. Supplementary Figure S10: subgroup analysis of pain intensity at rest at 12 h postoperatively by administration times. Supplementary Figure S11: subgroup analysis of pain intensity at rest at 24 h postoperatively by administration times. Supplementary Figure S12: subgroup analysis of pain intensity at rest at 48 h postoperatively by administration times. Supplementary Figure S13: subgroup analysis of pain intensity during mobilization at 6 h postoperatively by administration times. Supplementary Figure S14: subgroup analysis of pain intensity during mobilization at 12 h postoperatively by administration times. Supplementary Figure S15: subgroup analysis of pain intensity during mobilization at 24 h postoperatively by administration times. Supplementary Figure S16: subgroup analysis of cumulative opioid consumption in the first 12 h postoperatively by ages. Supplementary Figure S17: subgroup analysis of cumulative opioid consumption in the first 24 h postoperatively by ages. Supplementary Figure S18: subgroup analysis of cumulative opioid consumption in the first 48 h postoperatively by ages. Supplementary Figure S19: subgroup analysis of cumulative opioid consumption in the first 12 h postoperatively by administration times. Supplementary Figure S20: subgroup analysis of cumulative opioid consumption in the first 24 h postoperatively by administration times. Supplementary Figure S21: subgroup analysis of cumulative opioid consumption in the first 48 h postoperatively by administration times. Supplementary Figure S22: subgroup analysis of the incidence of PONV by ages. Supplementary Figure S23: subgroup analysis of the incidence of PONV by administration times.
