# Supplementary material for: Perioperative Low-Dose Ketamine for Postoperative Pain Management in Spine Surgery: A Systematic Review and Meta-Analysis of Randomized Controlled Trials
Source: Pain Res Manag. 2022 Mar 31;2022:1507097. doi: 10.1155/2022/1507097 (PMC8989618; doi:10.1155/2022/1507097)
Supplement: Supplementary Materials — Supplementary Figure S1: subgroup analysis of pain intensity at rest at 6 h postoperatively by ages. Supplementary Figure S2: subgroup analysis of pain intensity at rest at 12 h postoperatively by ages. Supplementary Figure S3: subgroup analysis of pain intensity at rest at 24 h postoperatively by ages. Supplementary Figure S4: subgroup analysis of pain intensity at rest at 48 h postoperatively by ages. Supplementary Figure S5: subgroup analysis of pain intensity during mobilization at 6 h postoperatively by ages. Supplementary Figure S6: subgroup analysis of pain intensity during mobilization at 12 h postoperatively by ages. Supplementary Figure S7: subgroup analysis of pain intensity during mobilization at 24 h postoperatively by ages. Supplementary Figure S8: subgroup analysis of pain intensity during mobilization at 48 h postoperatively by ages. Supplementary Figure S9: subgroup analysis of pain intensity at rest at 6 h postoperatively by administration times. Supplementary Figure S10: subgroup analysis of pain intensity at rest at 12 h postoperatively by administration times. Supplementary Figure S11: subgroup analysis of pain intensity at rest at 24 h postoperatively by administration times. Supplementary Figure S12: subgroup analysis of pain intensity at rest at 48 h postoperatively by administration times. Supplementary Figure S13: subgroup analysis of pain intensity during mobilization at 6 h postoperatively by administration times. Supplementary Figure S14: subgroup analysis of pain intensity during mobilization at 12 h postoperatively by administration times. Supplementary Figure S15: subgroup analysis of pain intensity during mobilization at 24 h postoperatively by administration times. Supplementary Figure S16: subgroup analysis of cumulative opioid consumption in the first 12 h postoperatively by ages. Supplementary Figure S17: subgroup analysis of cumulative opioid consumption in the first 24 h postoperatively by ages. Supplementary Figure S18: subgro [file 1507097.f1.pdf]

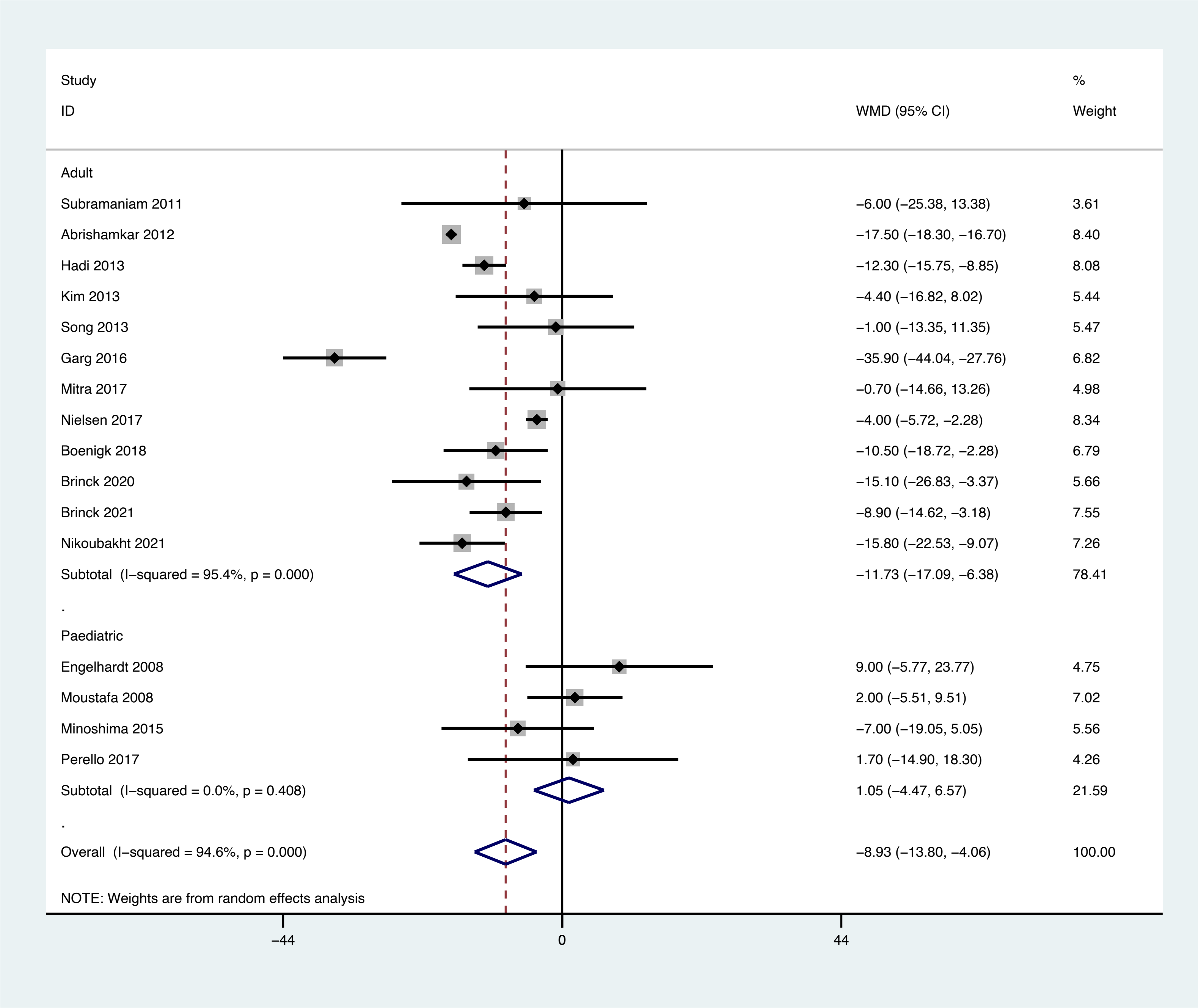

Figure 1. Subgroup analysis of pain intensity at rest at 6 h postoperatively by ages

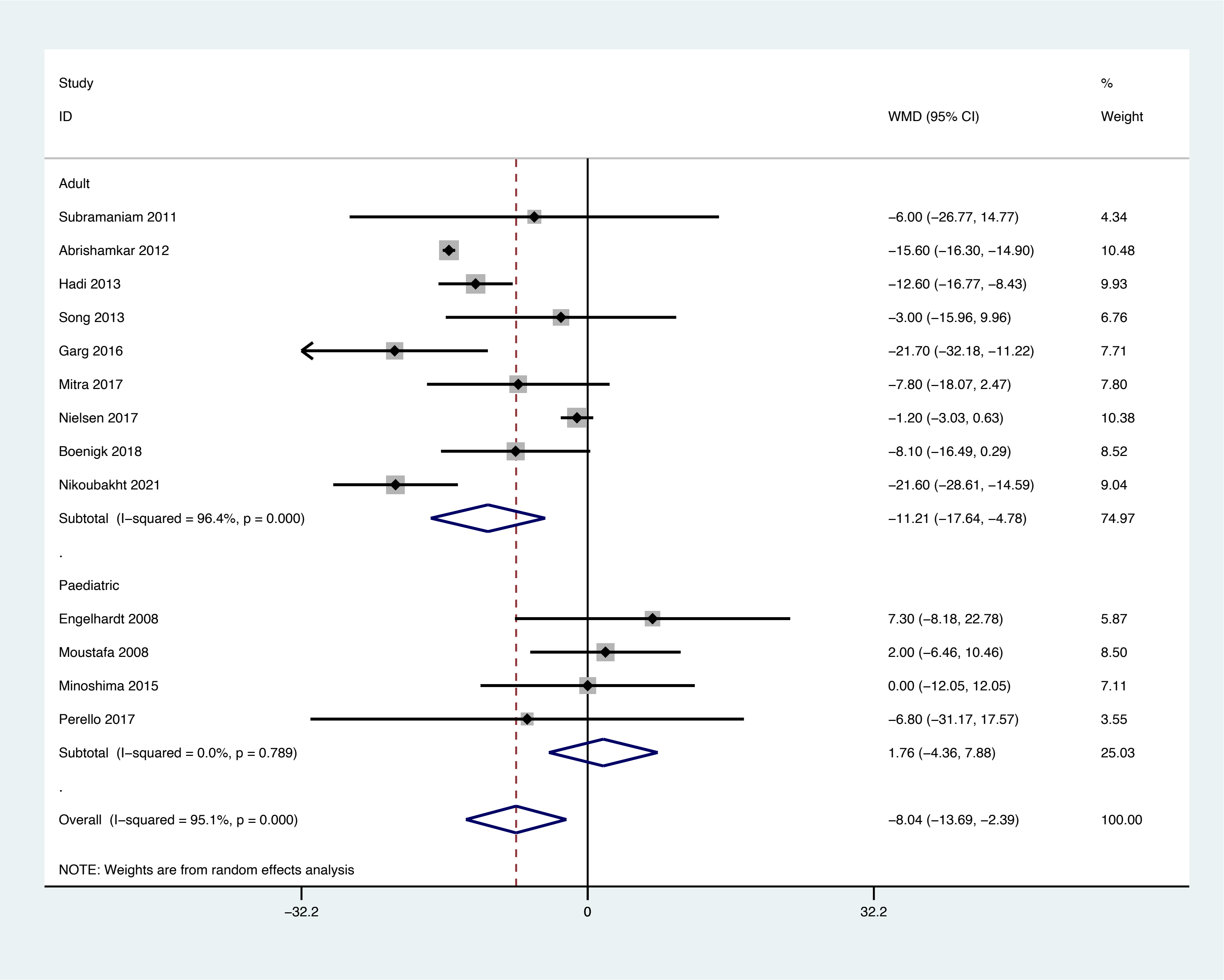

Figure 2. Subgroup analysis of pain intensity at rest at 12 h postoperatively by ages

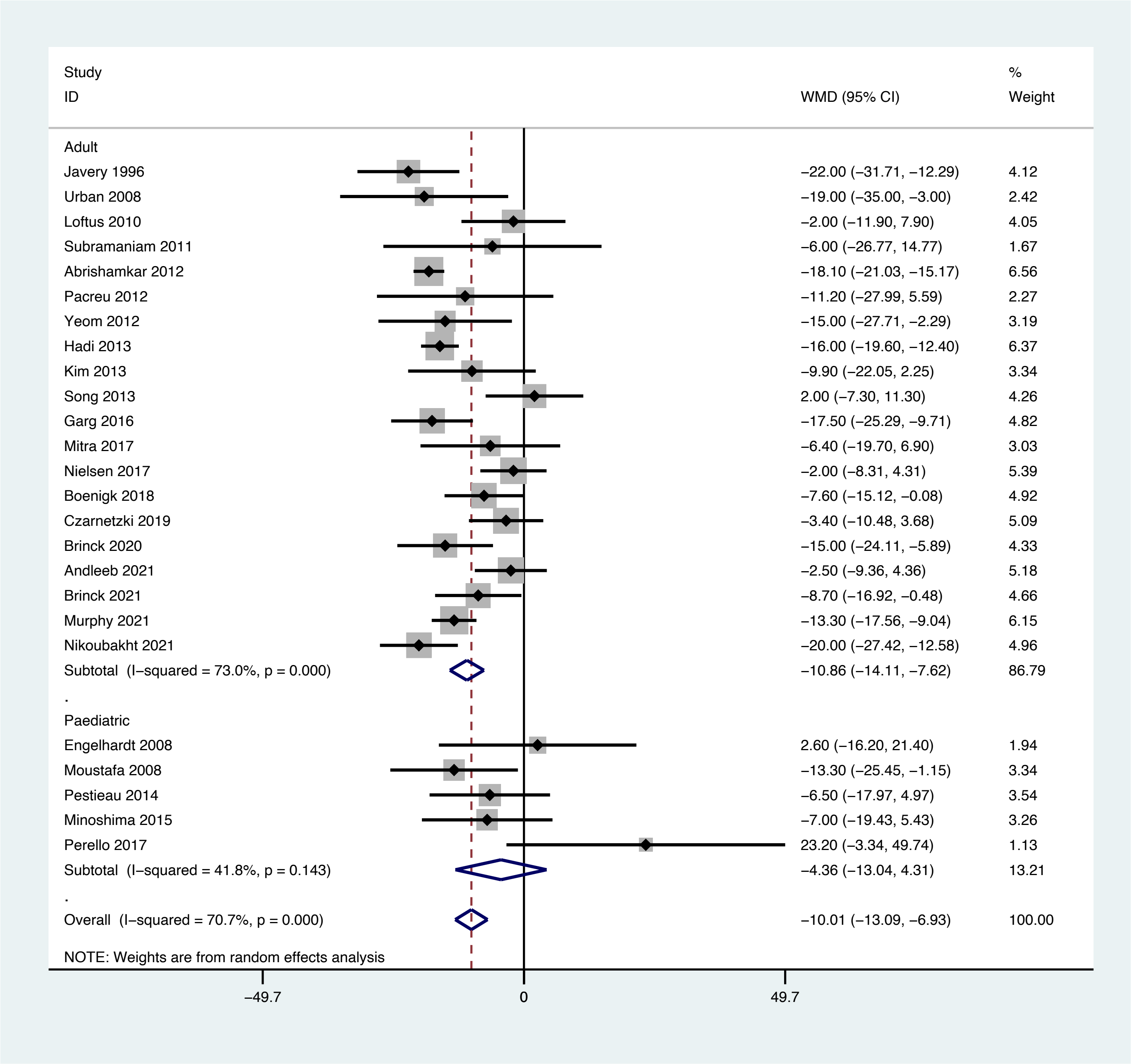

Figure 3. Subgroup analysis of pain intensity at rest at 24 h postoperatively by ages

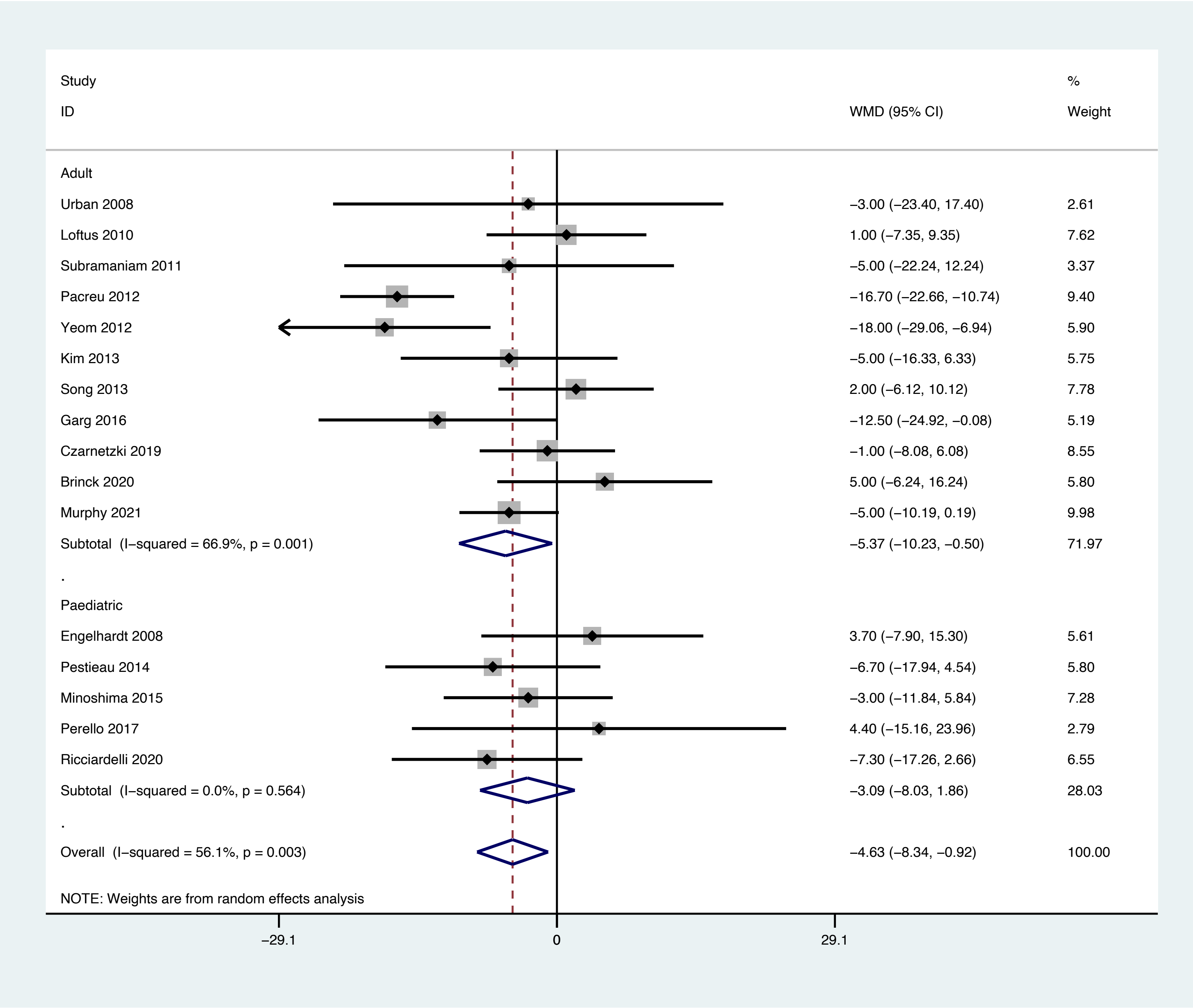

Figure 4. Subgroup analysis of pain intensity at rest at 48 h postoperatively by ages

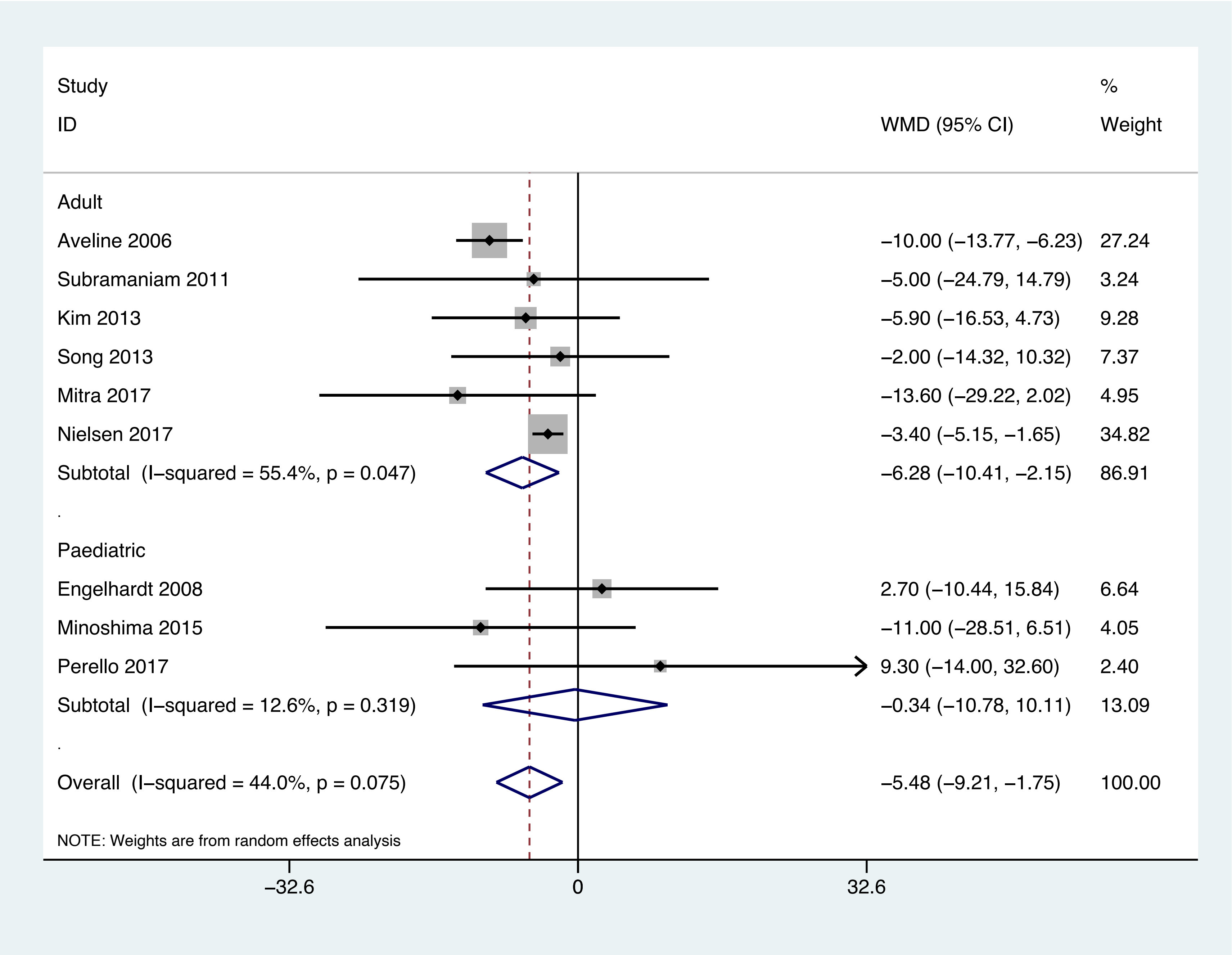

Figure 5. Subgroup analysis of pain intensity during mobilization at 6 h postoperatively by ages

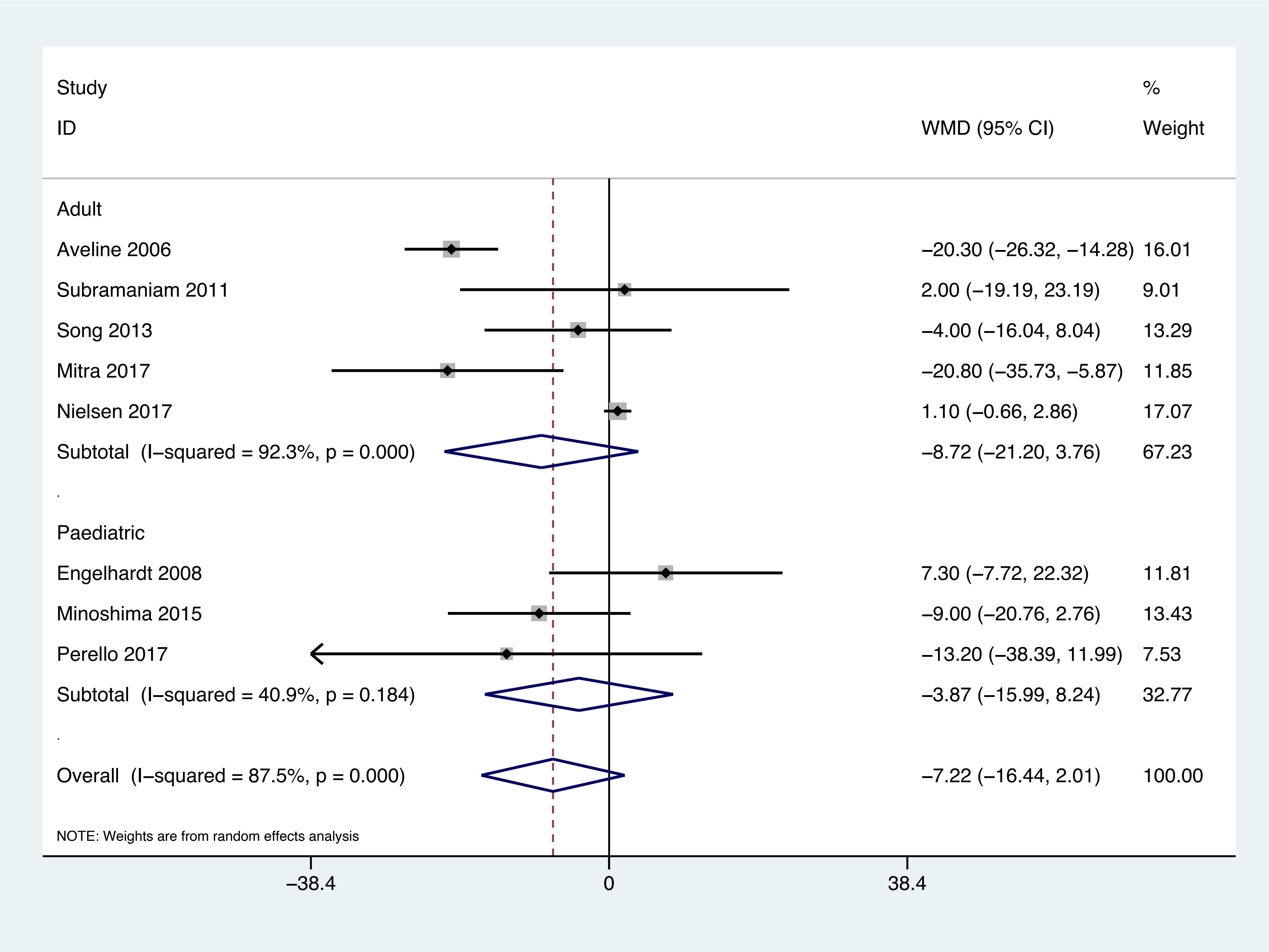

Figure 6. Subgroup analysis of pain intensity during mobilization at 12 h postoperatively by ages

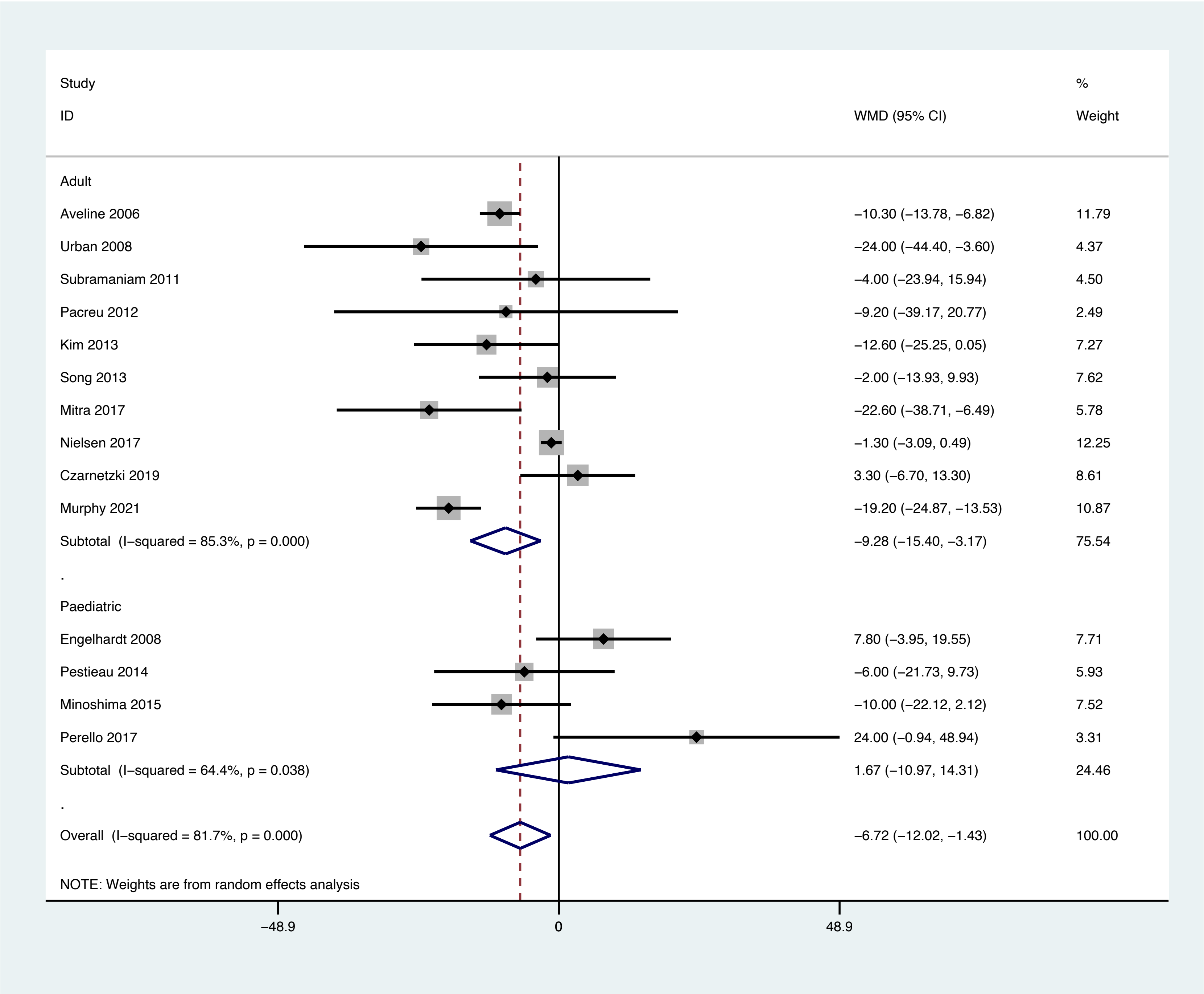

Figure 7. Subgroup analysis of pain intensity during mobilization at 24 h postoperatively by ages

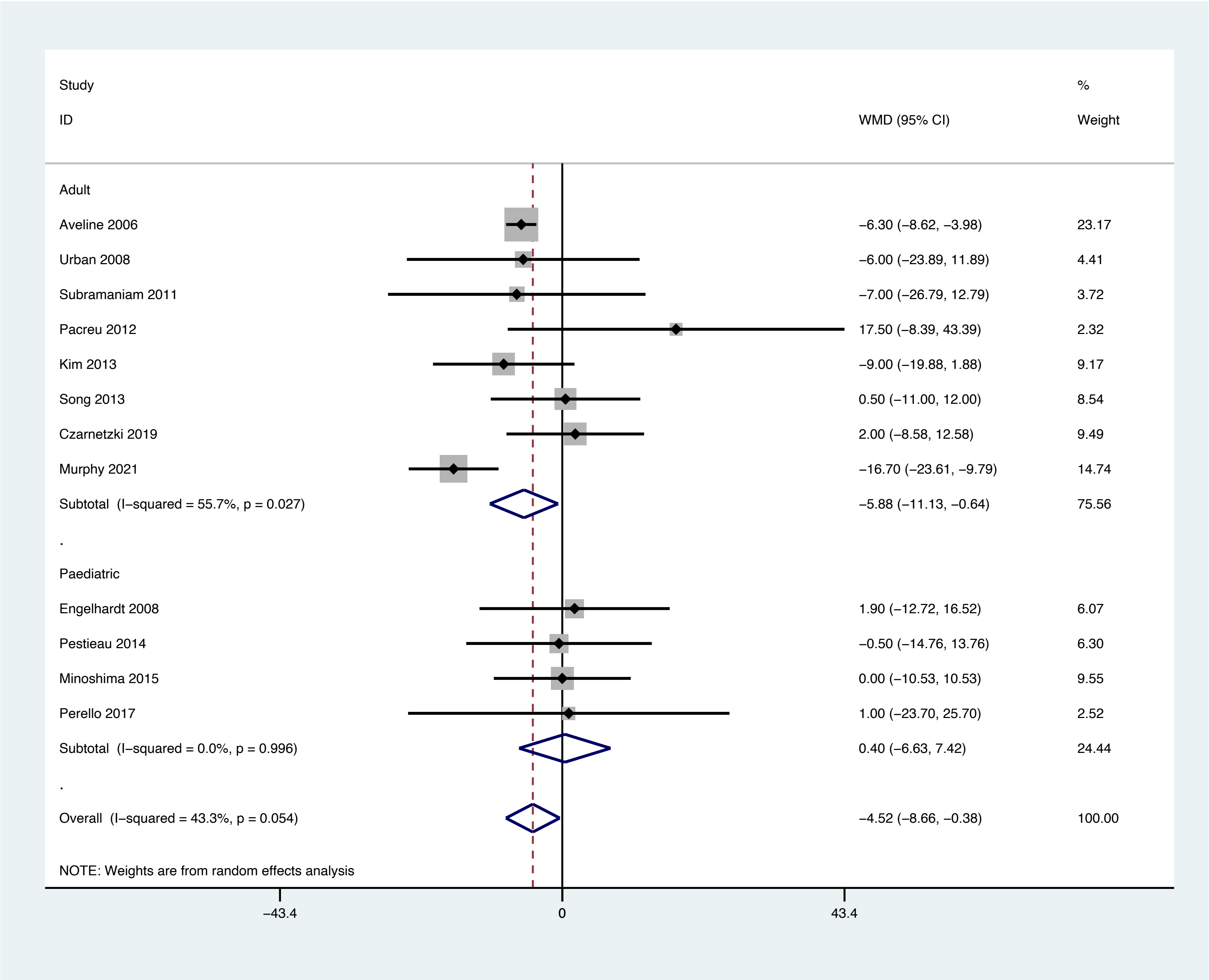

Figure 8. Subgroup analysis of pain intensity during mobilization at 48 h postoperatively by ages

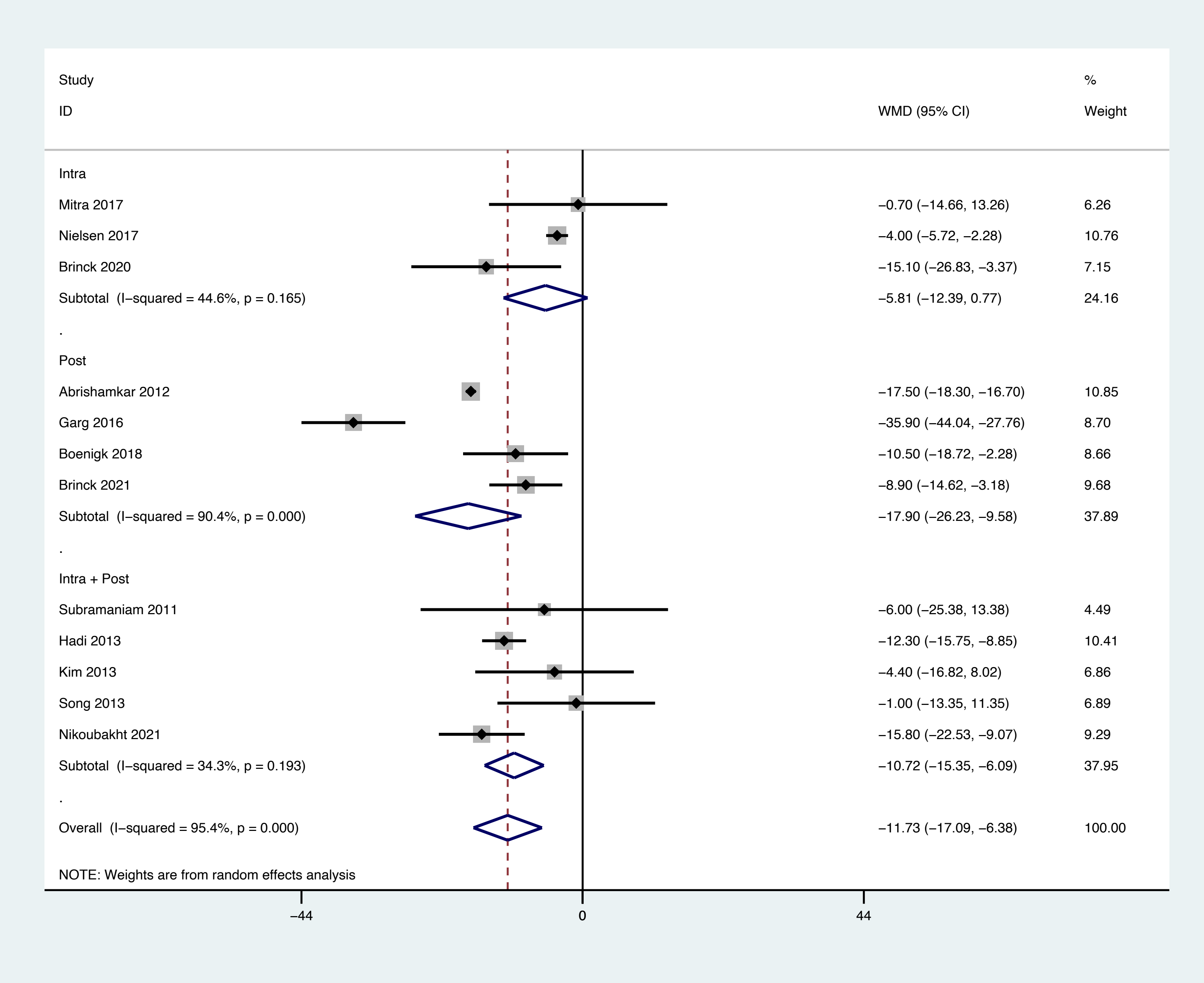

Figure 9. Subgroup analysis of pain intensity at rest at 6 h postoperatively by administration times

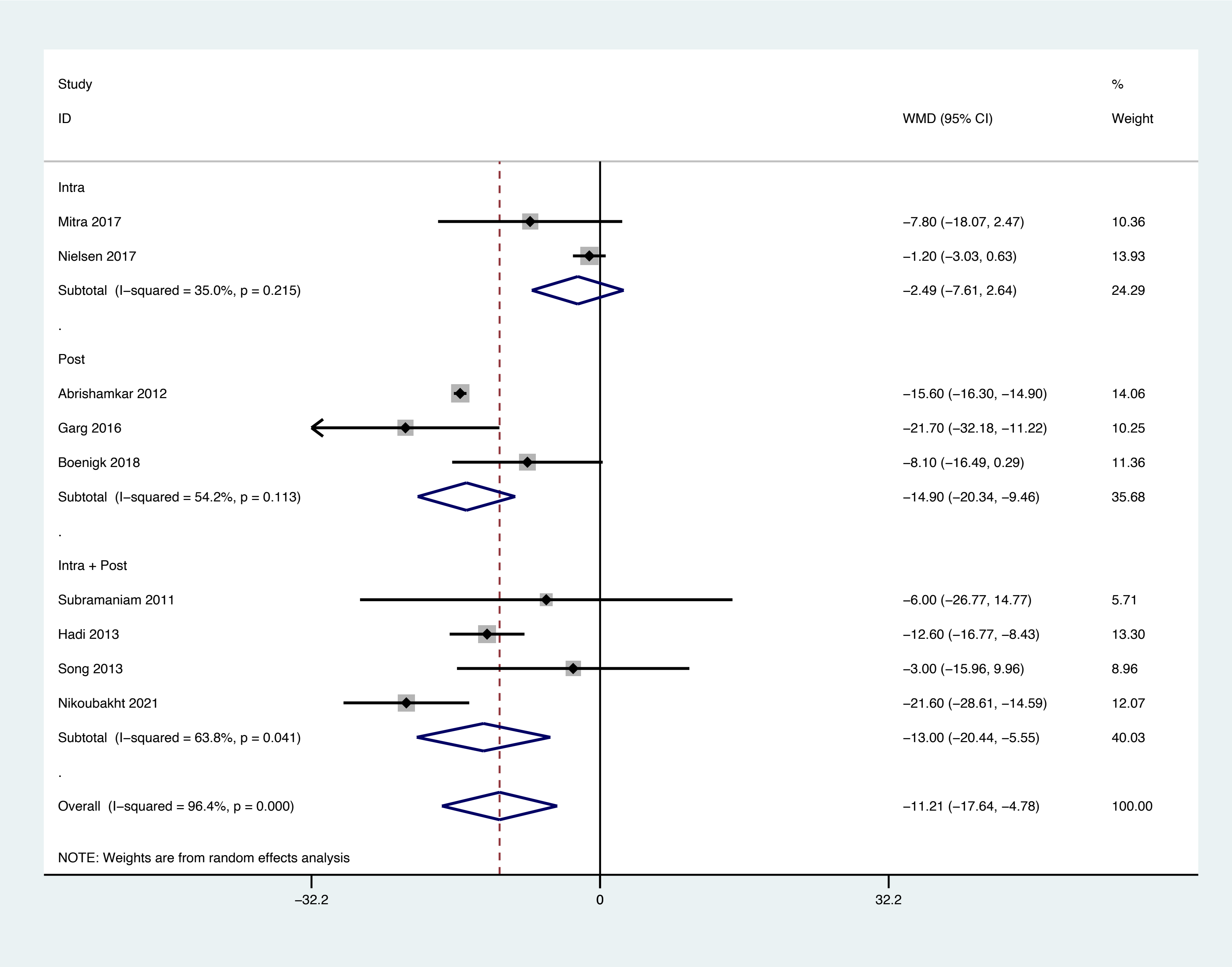

Figure 10. Subgroup analysis of pain intensity at rest at 12 h postoperatively by administration times

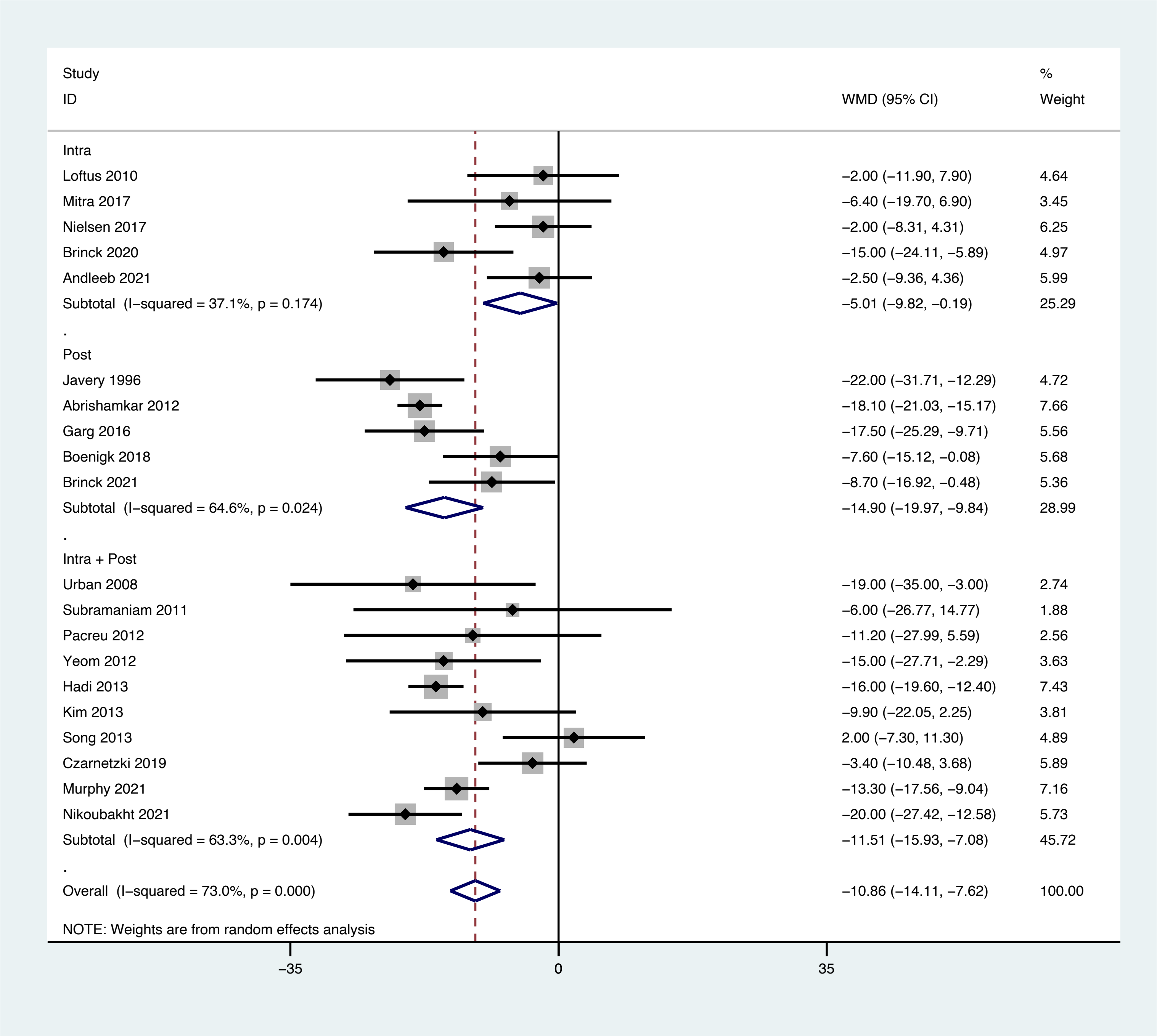

Figure 11. Subgroup analysis of pain intensity at rest at 24 h postoperatively by administration times

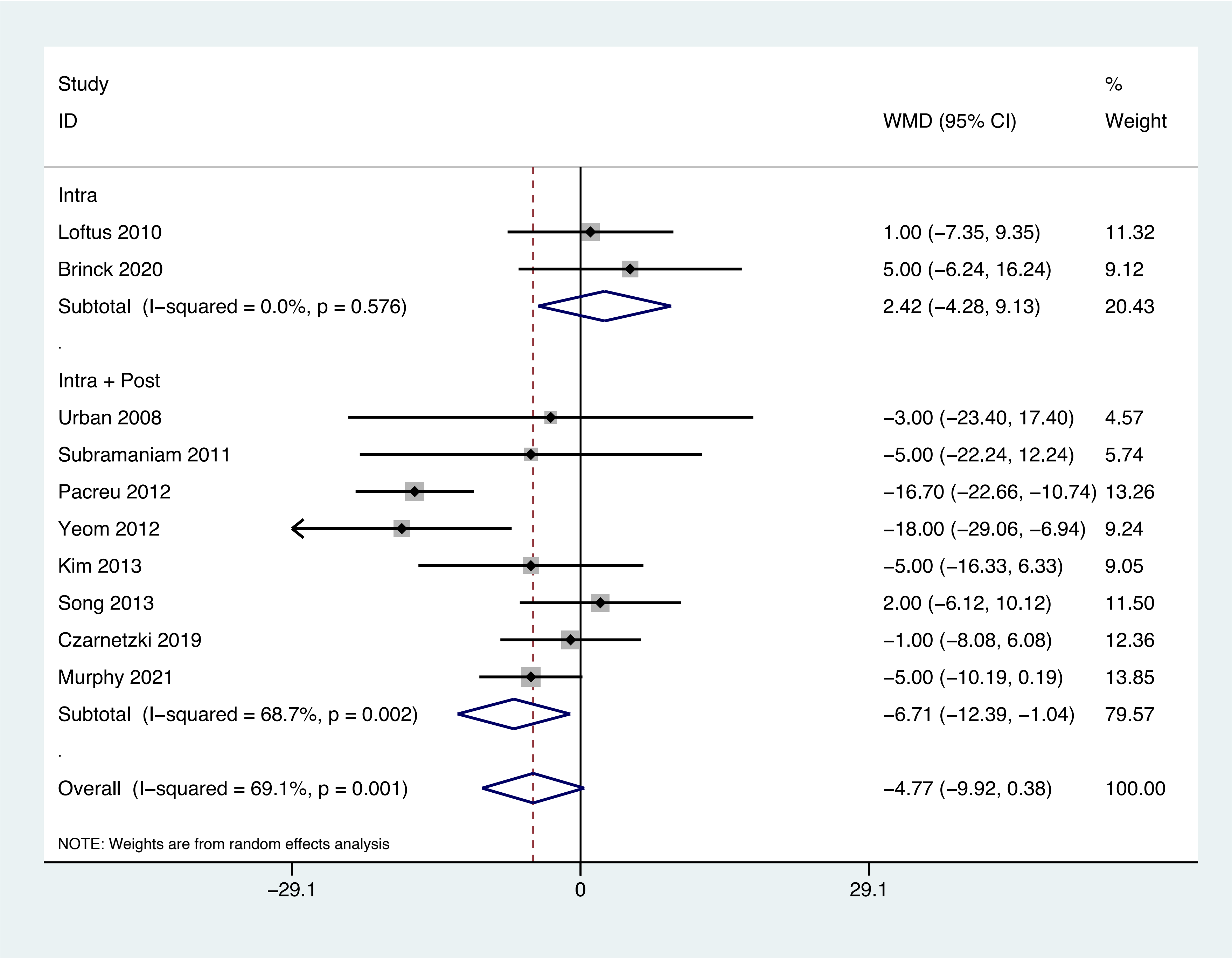

Figure 12. Subgroup analysis of pain intensity at rest at 48 h postoperatively by administration times

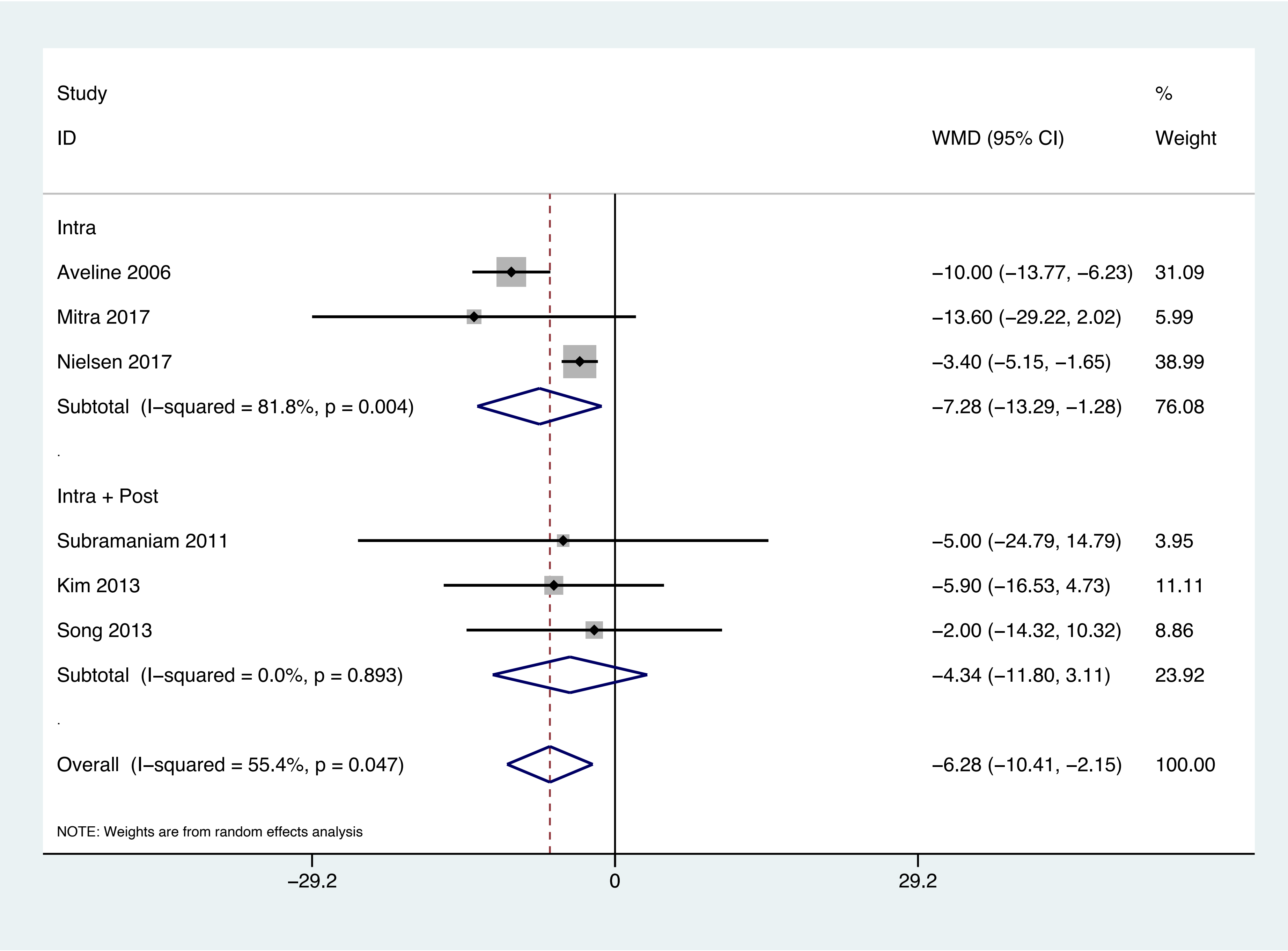

Figure 13. Subgroup analysis of pain intensity during mobilization at 6 h postoperatively by administration times

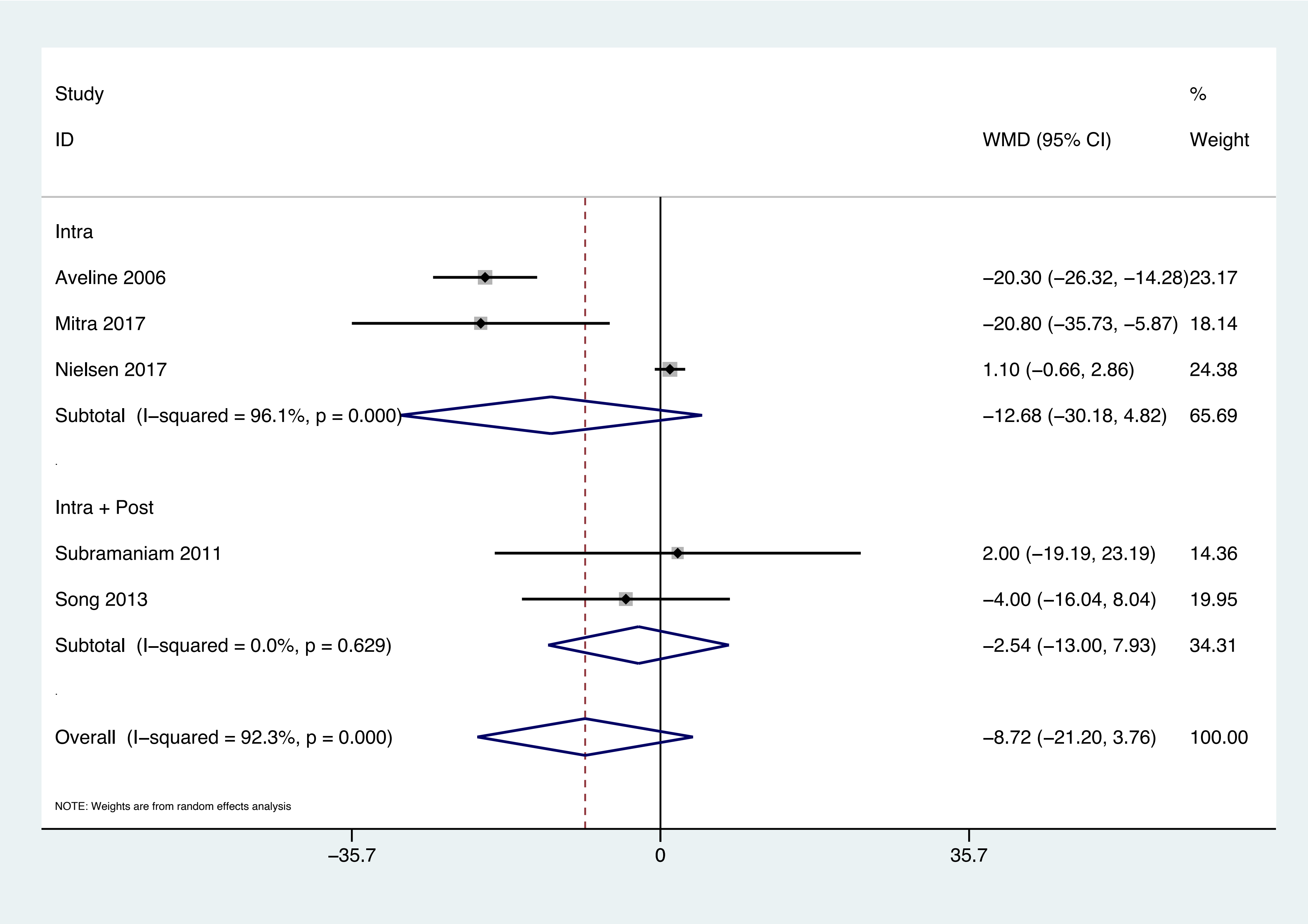

Figure 14. Subgroup analysis of pain intensity during mobilization at 12 h postoperatively by administration times

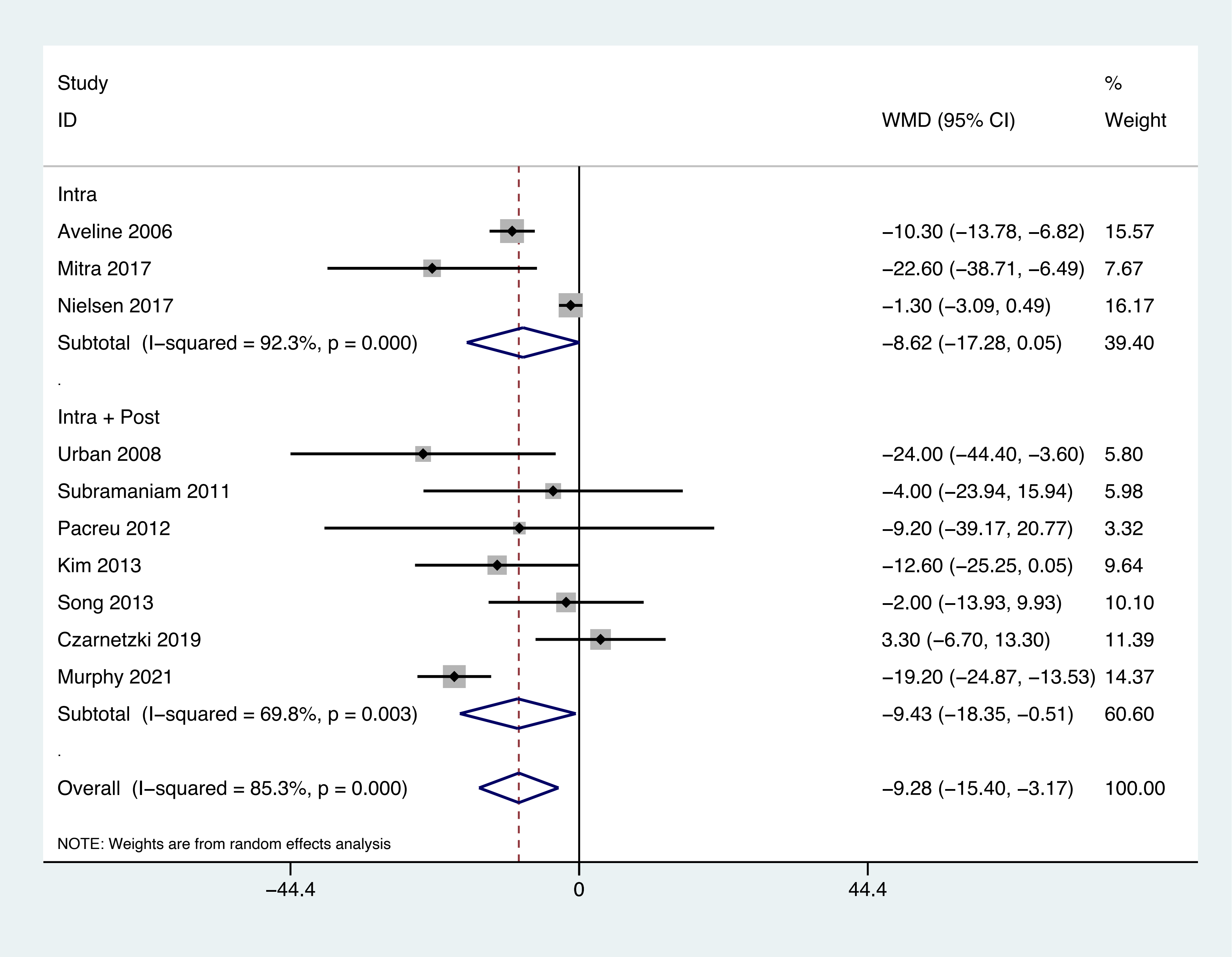

Figure 15. Subgroup analysis of pain intensity during mobilization at 24 h postoperatively by administration times

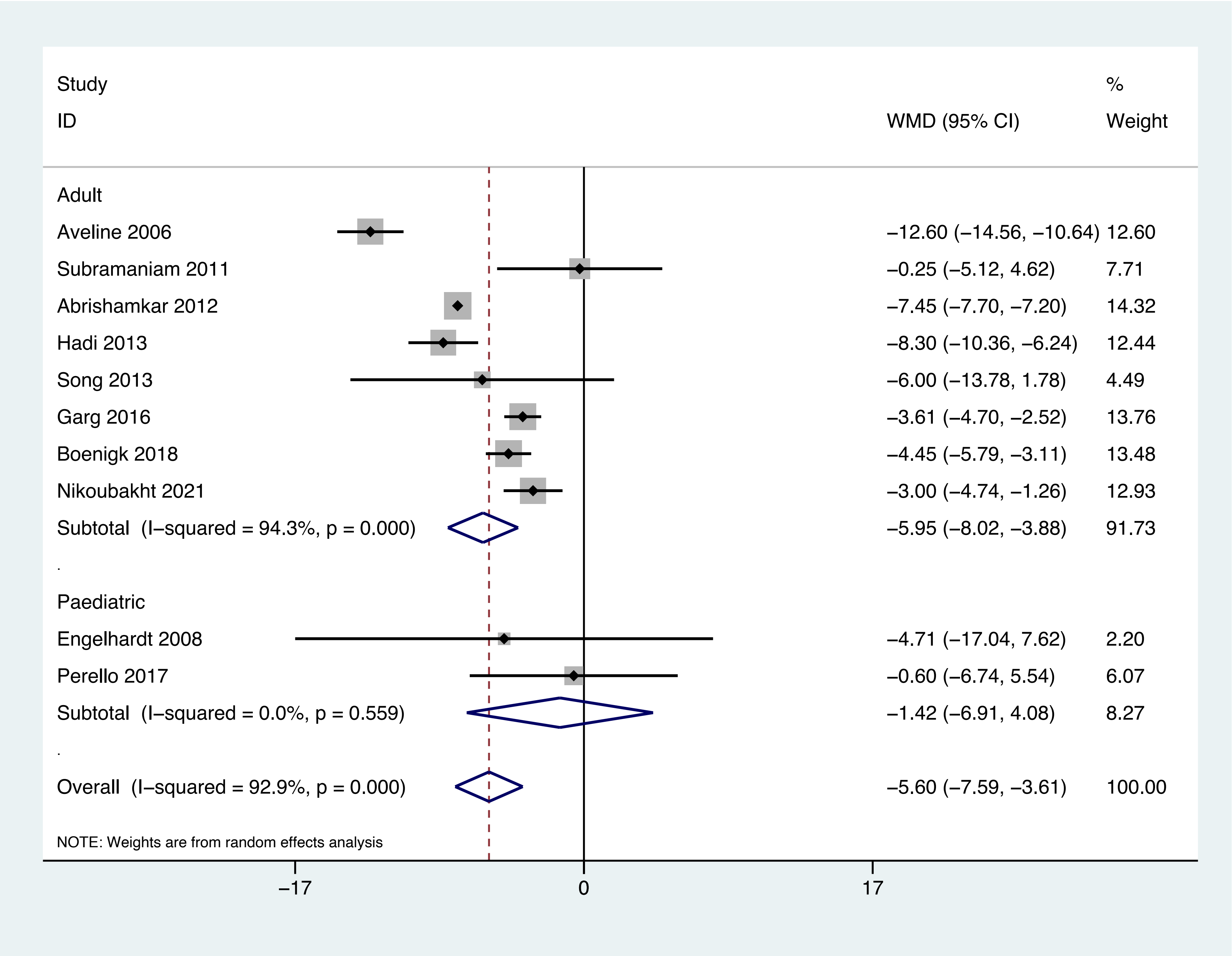

Figure 16. Subgroup analysis of cumulative opioid consumption in the first 12 h postoperatively by ages

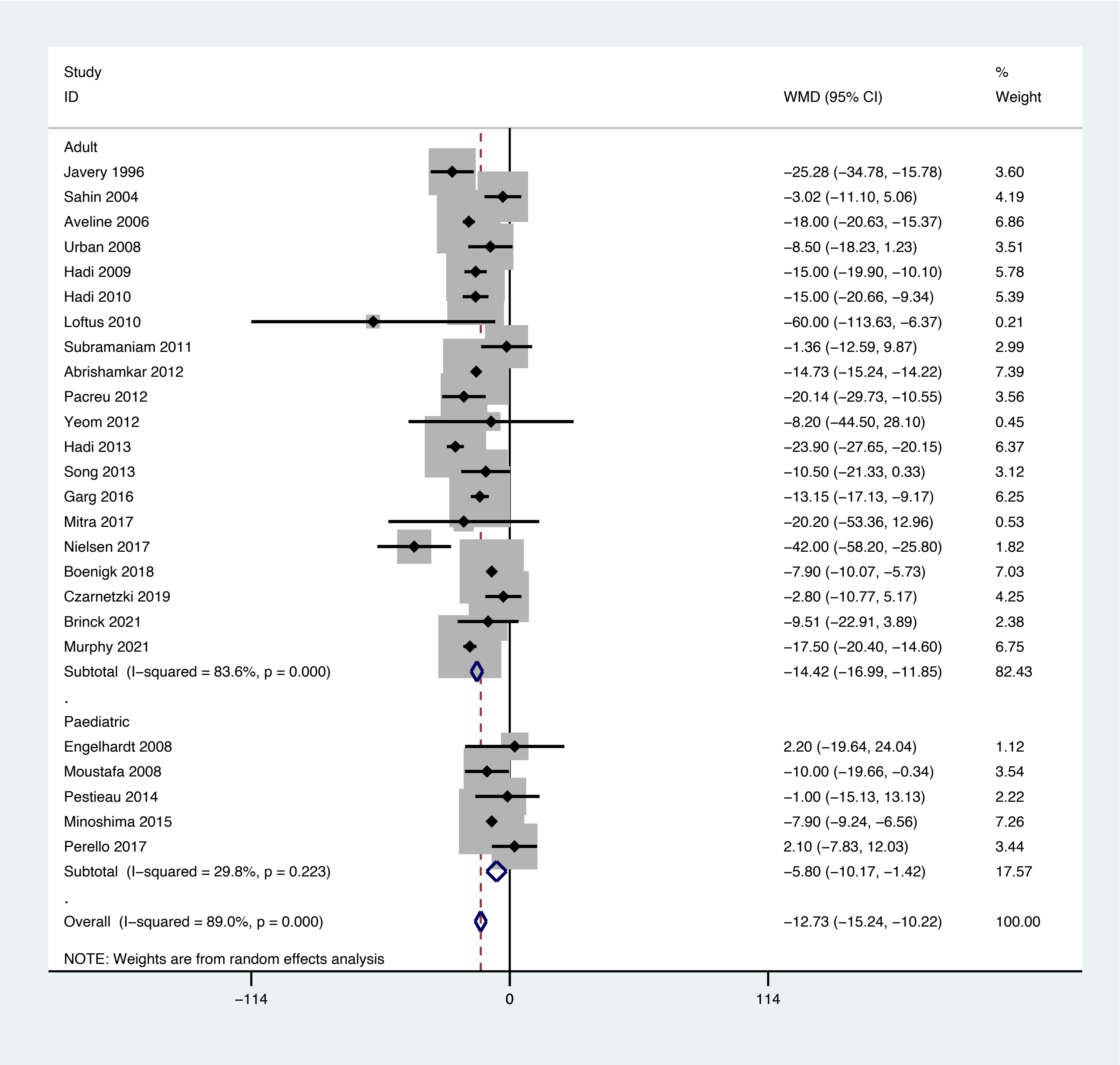

Figure 17. Subgroup analysis of cumulative opioid consumption in the first 24 h postoperatively by ages

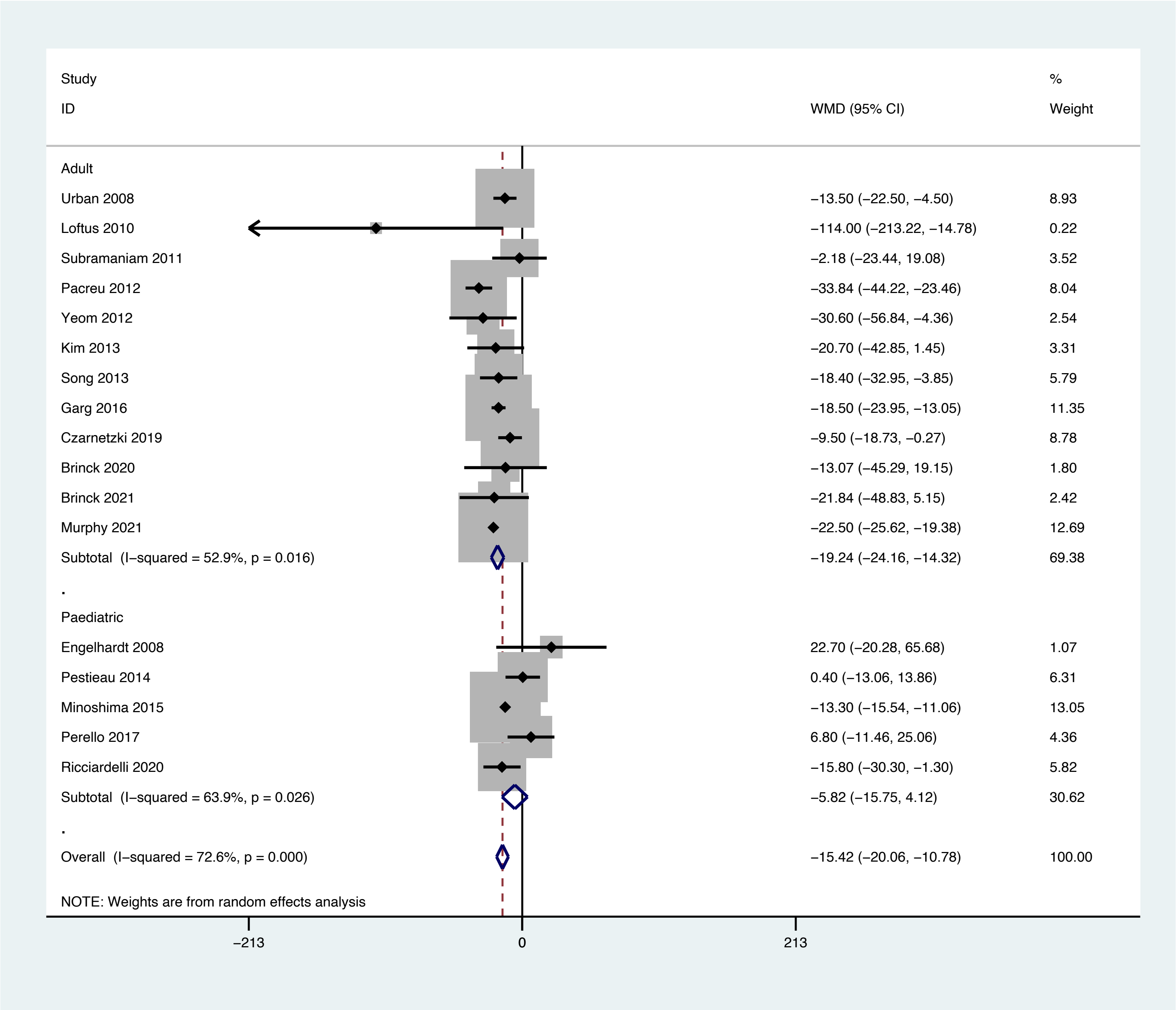

Figure 18. Subgroup analysis of cumulative opioid consumption in the first 48 h postoperatively by ages

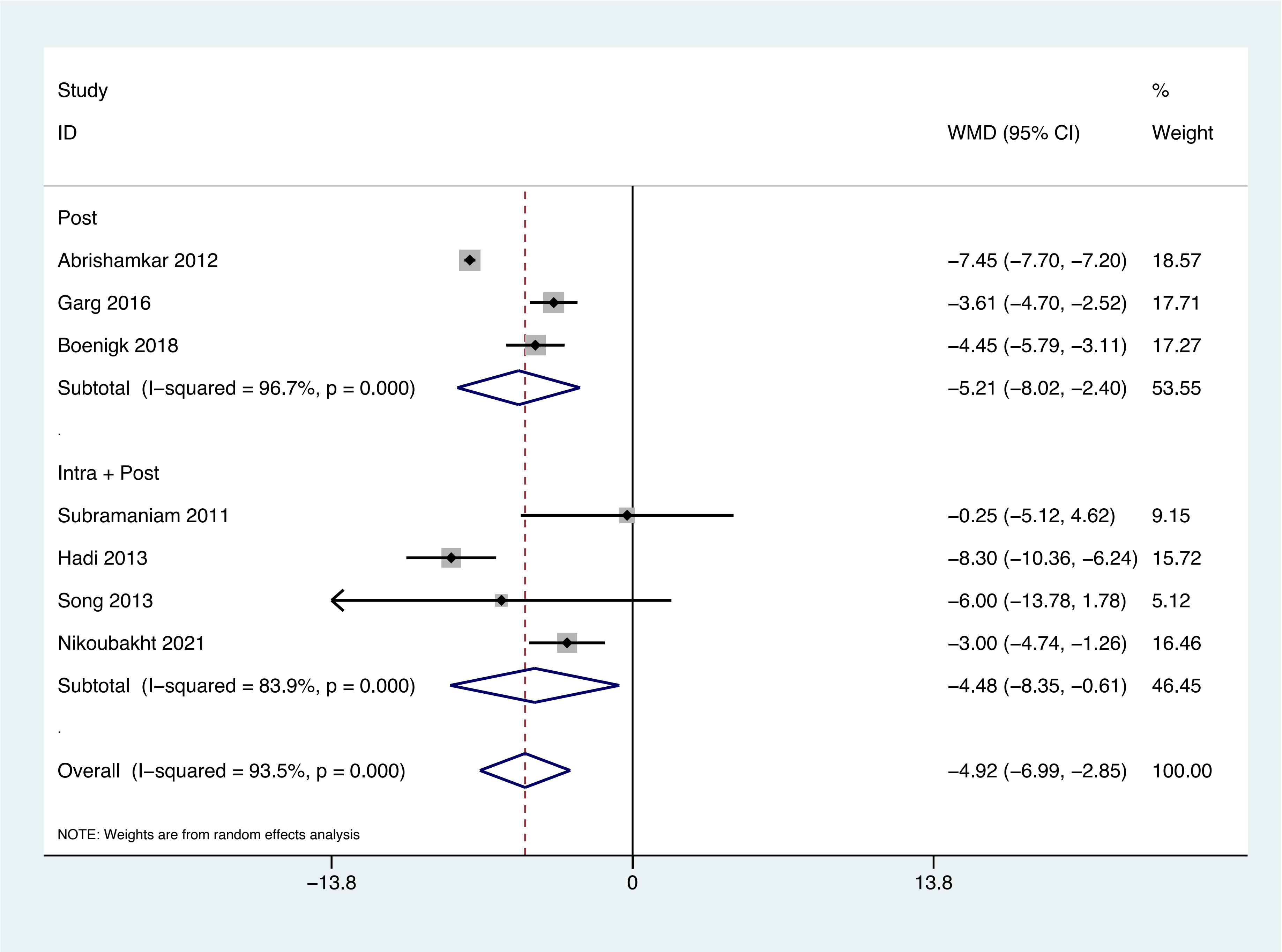

Figure 19. Subgroup analysis of cumulative opioid consumption in the first 12 h postoperatively by administration times

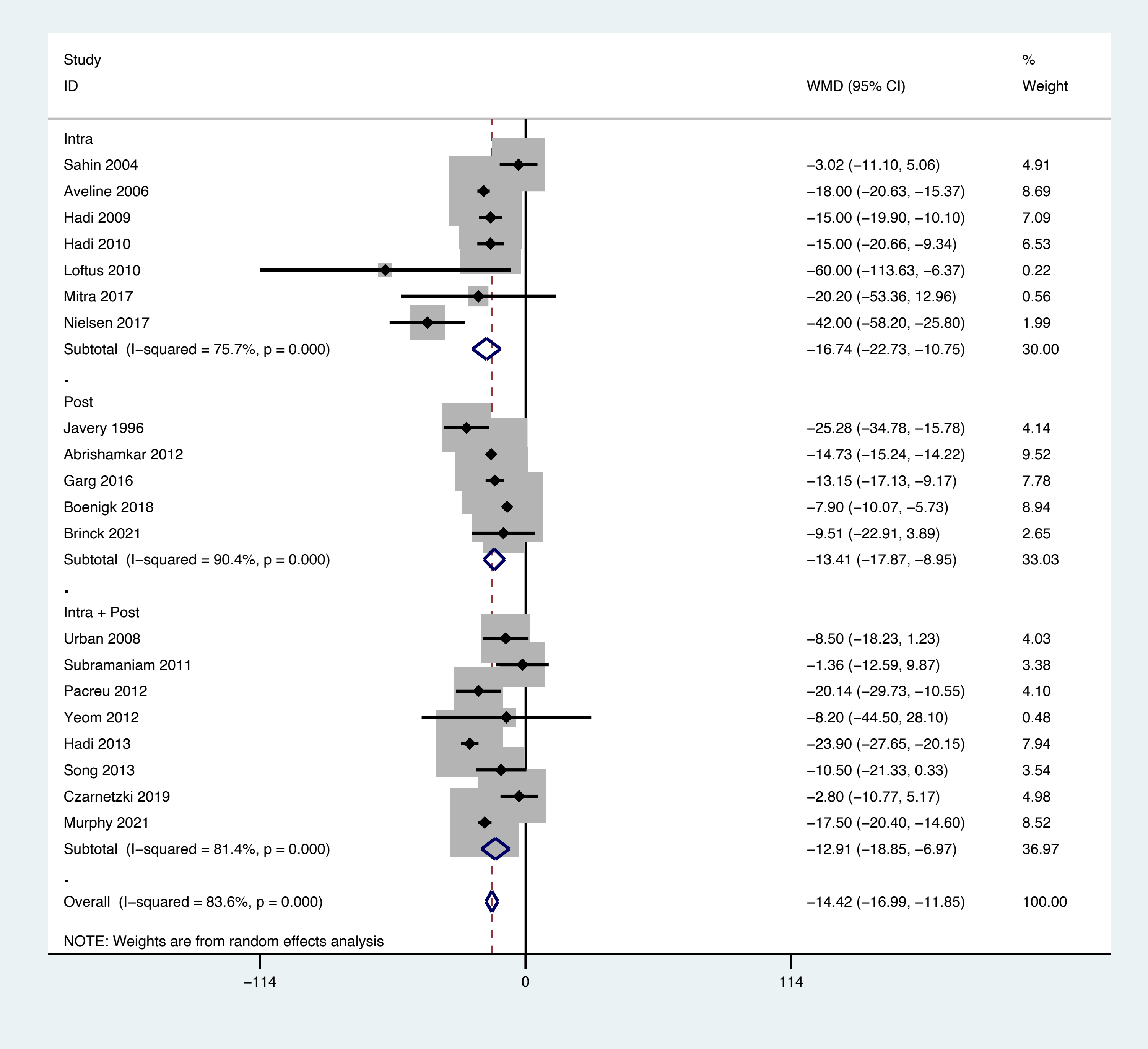

Figure 20. Subgroup analysis of cumulative opioid consumption in the first 24 h postoperatively by administration times

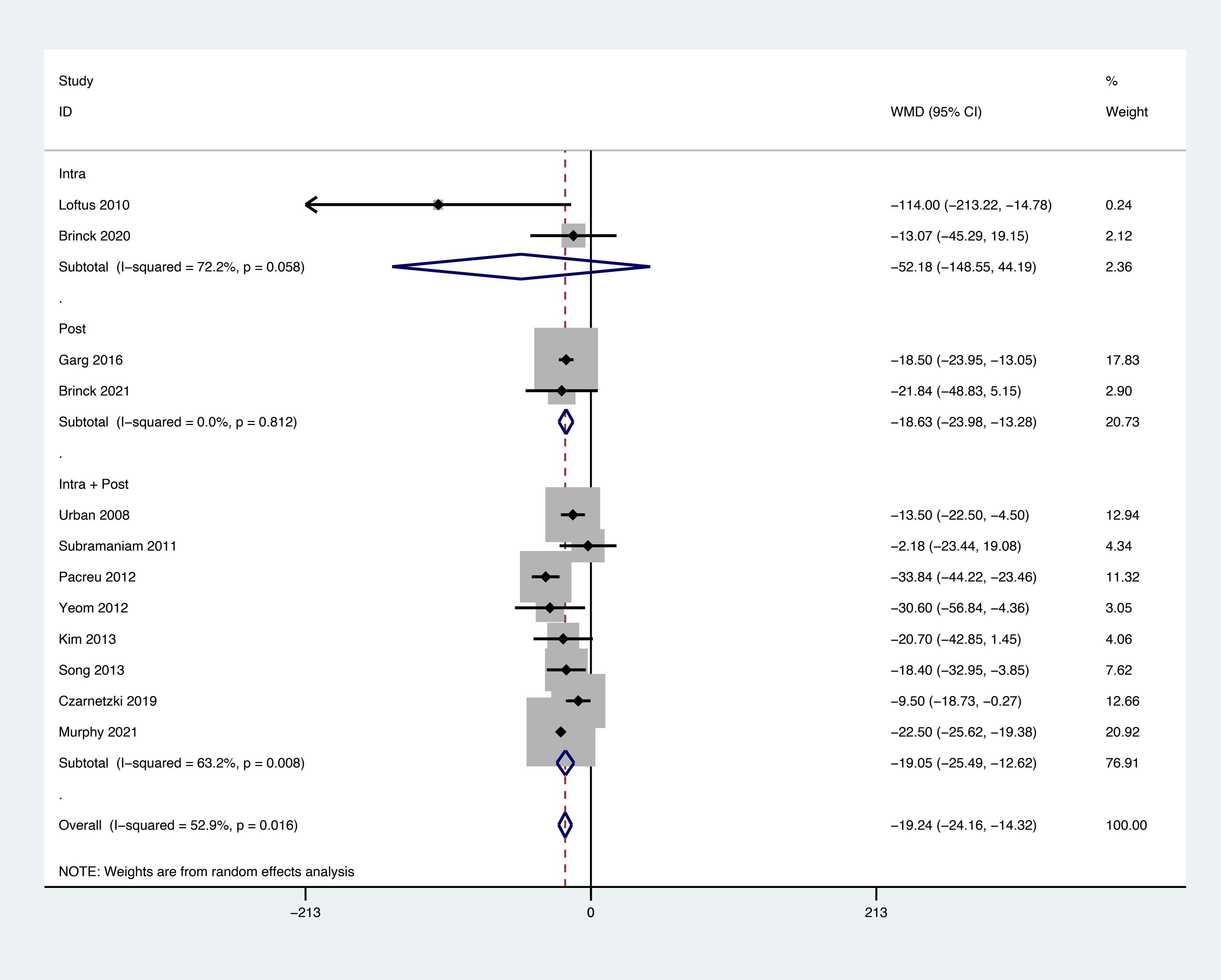

Figure 21. Subgroup analysis of cumulative opioid consumption in the first 48 h postoperatively by administration times

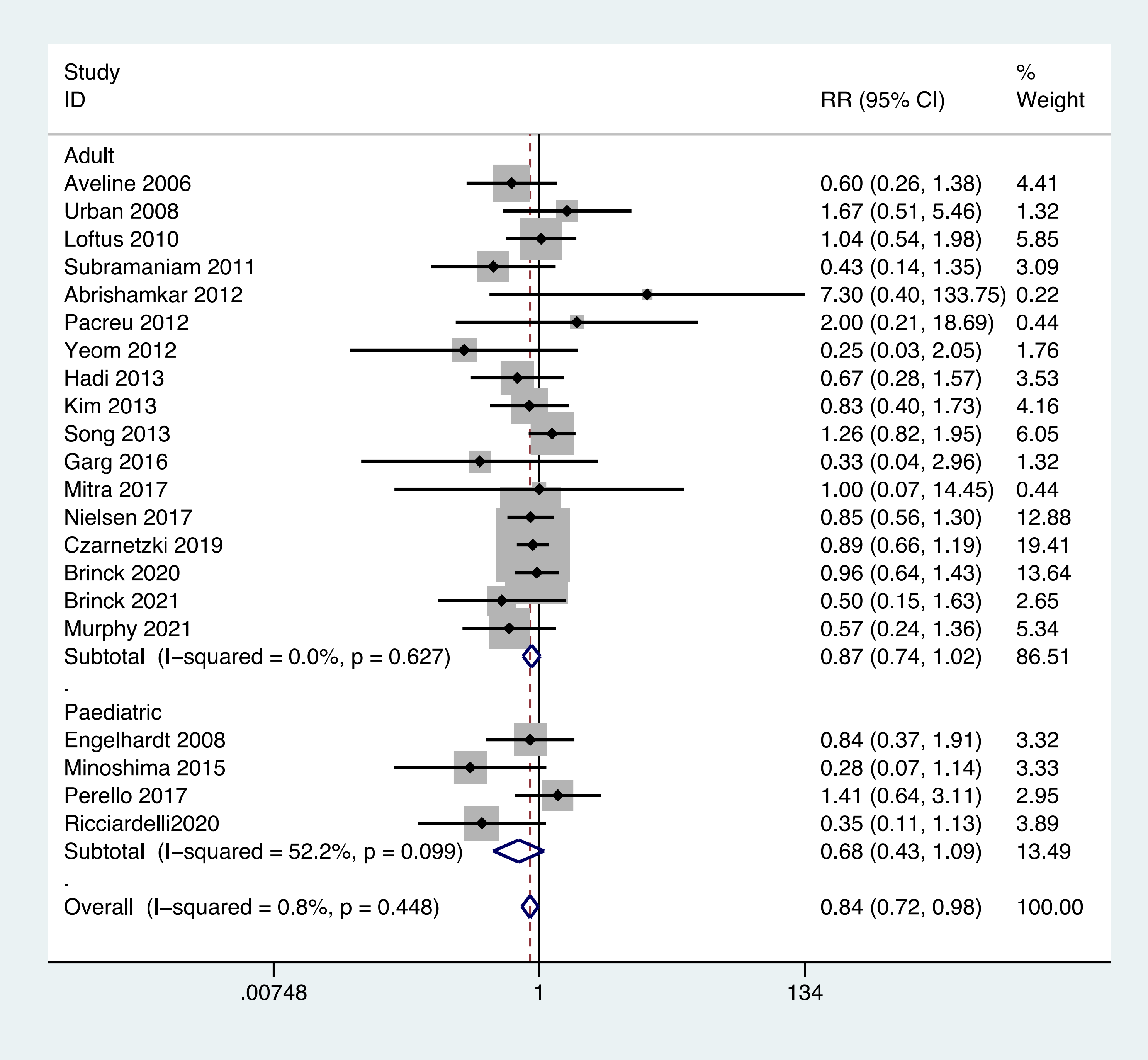

Figure 22. Subgroup analysis of the incidence of PONV by ages

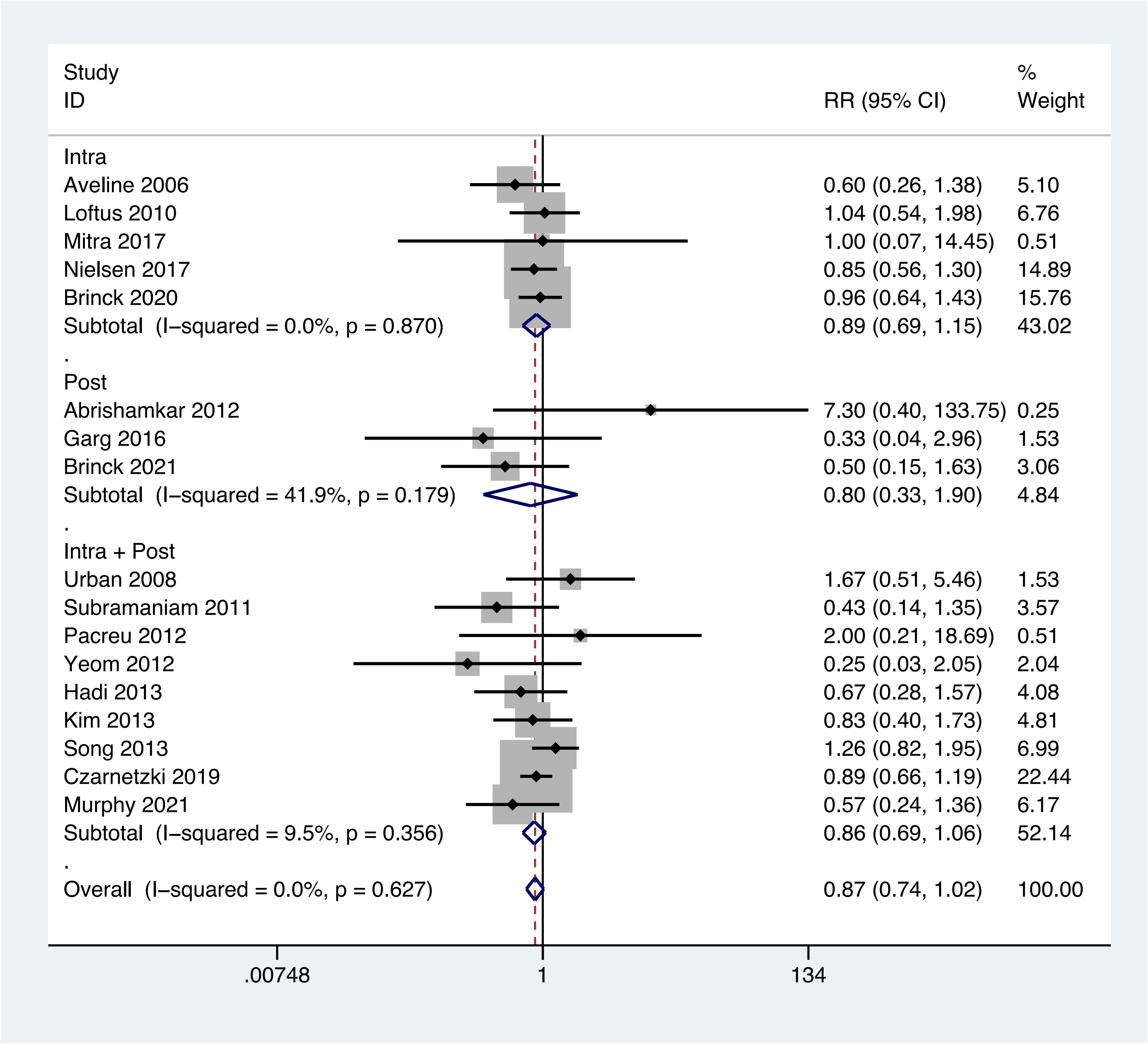

Figure 23. Subgroup analysis of the incidence of PONV by administration times
